# Women’s retention on the continuum of maternal care pathway in west Gojjam zone, Ethiopia: multilevel analysis

**DOI:** 10.1186/s12884-020-02953-5

**Published:** 2020-04-29

**Authors:** Amanu Aragaw Emiru, Getu Degu Alene, Gurmesa Tura Debelew

**Affiliations:** 1 Department of Reproductive Health and Population Studies, College of Medicine and Health Sciences, Bahir Dar University, Bahir Dar, Ethiopia; 2 Department of Epidemiology and Biostatistics, College of Medicine and Health Sciences, Bahir Dar University, Bahir Dar, Ethiopia; 3 Department of Population and Family Health, Faculty of Public Health, Institute of Health, Jimma University, Jimma, Ethiopia

**Keywords:** Maternal care, Antenatal, Birth, Postnatal, Continuum

## Abstract

**Background:**

The continuum of maternal care has been one of the effective approaches for improving the health of mothers and newborns. Although large numbers of Ethiopian women do not use maternal health services, points of drop out along the continuum are not understood well. Understanding of a particular point of maternal care dropout on the continuum, however, helps governments make effective interventions. This study aimed to assess the extent of women’s service utilization and the factors affecting retention on the continuum of care in West Gojjam Zone, Ethiopia.

**Methods:**

A community-based study linked to health facility data was conducted in June 2018. Data were obtained from 1281 mothers who gave birth to their last baby within the preceding 12 months from a two-stage cluster sampling. Data were collected via face-to-face interviews using a pretested questionnaire. Multilevel logistic regression models were used to examine the effects of individual and cluster-level factors on key elements of the continuum of care. The measure of fixed effects was expressed as Odds Ratio with 95% confidence interval.

**Results:**

The study revealed that only 12.1% of women completed the continuum of maternal care services (ANC4+, SBA, and PNC within 2 days after birth); while 25.1% of them did not receive any care during their recent births. There were commonalities and differences in the predictors of the three indicators of maternal health service utilization. Variables related to services received during antenatal care such as early initiation of ANC (AOR = 7.53, 95%CI, 2.94, 19.29) and receiving proper contents (AOR = 3.31, 95%CI, 1.08, 10.16) were among the predictors significantly associated with the completion of the continuum of care.

**Conclusions:**

The continuum of maternal care completion rate was extremely low, indicating that women were not getting the maximum possible health benefit from existing health services. The results also revealed that maternal health service utilization was influenced by factors operating at various levels-individual, household, community, and health facility. Since antenatal care is considered an entry point for the subsequent use of maternal services, strategies that aimed to improve maternal health service utilization should target early initiation and antenatal care quality.

## Background

Most of the time, motherhood is a source of joy to the family and women in particular. Yet, each pregnancy poses risks to women and newborns in low-income countries, particularly in Sub-Saharan Africa (SSA), where maternal services are limited [1]. It is evidenced that the lifetime risk of maternal death in SSA countries, on average, is about 1 in 36 compared to 1 in 4900 women in high-income countries [2]. Similarly is the case of child mortality, with globally 5.3 million deaths in children under 5 years in 2018, 1 out of 13 children in SSA die before celebrating their 5th birthday compared to 1 in 189 in developed countries [3].

The situation in Ethiopia is not exceptional; the high toll of maternal and child mortalities remains the single most important challenge to the health sector. At 412 deaths per 100,000 live births [4], Ethiopia’s maternal mortality ratio is one of the highest in the world. Ethiopia is also among the countries that contribute more than half of the global under-five deaths; the others being India, Nigeria, Pakistan, and the Democratic Republic of the Congo [3].

To date, many strategies have been implemented in an attempt to improve maternal health outcomes [2]. Of the strategies, the continuum of maternal care (CoC) has recently received an important recognition. The COC has been designed as a key program strategy for improving the health and wellbeing of women, and as a means to reduce the high toll of maternal morbidity and mortality [5, 6]. This program requires an integrated service delivery system that coordinates the key maternal services (from pre-pregnancy to delivery, and postnatal care) with a continuous stream of quality at each level [5].

The provision of antenatal services that are timely (first visit during the first trimester of pregnancy) [7, 8], frequent (four or more visits) [7, 9, 10], and adequate (with proper contents) [7, 9, 10] improve the uptake of subsequent maternal services. Skilled attendance at birth reduces deaths for both the mother and the newborn [9, 11]. Postnatal care, especially within the first 48 h after birth, has been essential to ensuring women’s and children’s health [9]. Postpartum family planning also reduces a woman’s reproductive risks associated with unintended pregnancy [5].

With regard to healthcare, Ethiopia has made some health reforms to carry out initiatives for safe motherhood. The government has tried to improve access to basic maternal health services through primary healthcare expansion and charge-free maternal health services [12]. To this effect, 16,440 health posts, 3547 health centers, and more than 311 hospitals have become functional [13]. Besides, the ministry of health has already set 34 priority interventions along the continuum of care [14].

In spite of these efforts, however, the use of maternal health care services along the entire cascade of maternity care (antenatal care, facility delivery, postnatal care, and family planning) is still very low compared to other Sub Saharan African (SSA) countries [15]. Currently, 22% of married women have an unmet need for family planning. Two- thirds of mothers do not receive the recommended number of antenatal care visits and many of them book late; while 72.6% of births take place at home; and only 16.5% of women have postnatal checks [14]. To improve the rate of service use across the continuum of care, factors influencing women’s service utilization need to be identified [1].

A body of literature examined the factors influencing women’s service utilization in Ethiopia [16–18]. However, many studies have treated antenatal, delivery, and postnatal care as separate entities either at the design and/or at the analysis stages. On the other hand, targeting each maternal service separately does not necessarily ensure that every woman receives a package of interventions continuously from pre-conception to post-delivery stages and beyond [5, 19]. Also, such an artificial division obscures the fact that pregnancy and post-pregnancy is a process, with each phase being critically affected by what preceded it [20].

In addition, the previous studies focused primarily on individual-level factors with little attention given to community factors and service delivery environment. However, this could underestimate the importance of considering contextual factors when designing appropriate maternal health strategies in the study area and the country at large. Moreover, while the multilevel modeling that controls the nesting effect of clusters at different levels is appropriate, the earlier studies have used the traditional logistic regression model that could not adequately handle hierarchical constructs.

Therefore, for bridging all those gaps in the contemporary empirical literature, this study examined the role of level 1 (individual and household-related factors) and level 2 (community and facility-level factors) on the utilization of the key elements of the continuum of maternal care using the multilevel logistic regression modeling.

## Methods

### Study setting

This study was conducted in the West Gojjam Zone of the Amhara region. The center of the Zone, Finote-Selam town, is located 395 km away from Addis Ababa‚ capital of Ethiopia. Administratively the zone comprises 13 rural districts and 2 town administrations with a total population of 2,611,925 people, of whom 615,892 (23.58%) were women in the reproductive age group [21].

Concerning the health care facilities in the zone, there were six public primary hospitals, 103 health centers, and over 374 health posts; all collectively being staffed by 51 medical doctors,227 public health officers, 1016 nurses, 293 midwives, 266 pharmacists and druggists, 200 laboratory professionals, and 850 health extension workers. In addition, there were 115 private health facilities (1general hospital and 114 clinics of different types) during the time of the survey [21].

### Study design and period

A community-based household survey linked to health facility data has been carried out in June 2018. In theory, linking household data about healthcare-seeking behavior of respondents to facility data regarding facility readiness is becoming an effective approach for improving coverage measurement [22].

### Study population and sampling

Women in the reproductive age group who gave birth in 12 months before the survey date were the study population**.** A multistage cluster sampling was employed to reach the study population. For this, the study area was first stratified into 13 rural districts and 2 town administrations (Finote-Selam and Burie towns). Of these, 5 districts (4 rural districts and 1 town administration) were selected randomly. Second, 13 Kebeles (clusters now onwards) were selected using simple random sampling. Then, all women who met the inclusion criteria in the selected kebeles were included in the study. The list of all births for rural women was obtained from the family folder of health extension workers while a preliminary census was conducted before the actual data collection in the urban setting of the selected kebeles.

Both the single population and double population formulas were considered in computing different sample sizes. For the single population formula the following assumptions were considered: 95% confidence level, 4% margin of error, 16.5% proportion of PNC utilization [14], design effect of 2, and 10% non-response rate. For the latter, the required sample size was done through the STAT CALC program of the Epi-Info statistical package V.7.0 and the following assumptions were considered: 5% level of significance (two-sided); 90% power; 1:3 ratio of maternity service users to non-users, design effect of 2, and 10% non-response rate. Of the different samples computed in this study, the largest sample size (that is 1294) was obtained from the single population formula. During the time of data collection, however, 1337 women who met the inclusion criteria were included in this study, while 1281 women responded to the questions.

Furthermore, during the house-to-house survey public health facilities (hospitals and health centers) providing at least basic maternal health services for that community were identified. To this end, all eligible public hospitals and health centers that deemed functional at least for a year before the survey were part of the facility survey. As a result, the survey included 15 public health facilities in all five districts.

### Variables and measurements

#### Outcome variables

Retention on the continuum of maternal care services at three levels: I) ANC1 to ANC 4+; II) ANC 4+ to SBA, and III) from SBA to PNC within 48 h after birth. These combined indicators were dichotomized to construct three binary variables (one for each outcome variable), “1” if the services were received, and “0” otherwise.

#### Outcome variable 1

Compares women who received four or more antenatal care visits against those who received less than four visits. Accordingly, the outcome was coded “1” if a woman received at least four ANC visits and “0” otherwise. **Outcome variable 2:** about the continuity of care from four or more antenatal care to skilled delivery. Hence, this variable was coded ‘1’ if a woman had at least four ANC follow-ups and facility delivery, and coded “0” when a woman made at least four antenatal care visits but failed to attend facility delivery. **Outcome variable 3:** This is about the continuity of care from ANC to SBA and PNC within the first 48 h after birth. The two categories of the outcome variable were “1” if a woman received four or more antenatal care visits, skilled birth attendance, and postnatal care within 48 h (that is completing the continuum of care), and coded “0” if she received at least four antenatal visits and skilled birth attendance but failed to attend PNC within 48 h after birth.

#### Independent variables

Ranges of individual, household, community, and facility-level variables have been selected based on their theoretical and empirical relevance applied in different kinds of literature, and they were grouped into two levels. **Level 1 variables (lower-level variables**) included individual and household related factors such as age, educational status of women and partners, occupation of both partners, birth order, intendedness of the pregnancy, wealth index, previous obstetrical history, timing and contents of antenatal care, and mode of delivery. **Level 2 variables (higher-level variables)** included aggregation of community factors (place of residence and distance to health facility) and health facility variables (health facility to population ratio and level of readiness of healthcare facilities.

In the household surveys, women who already had any antenatal care follow-ups were asked about the basic components that the WHO has determined essential for every pregnant woman [7]. The components included body weight, blood pressure, urine analysis, blood testing, tetanus toxoid injection, deworming, iron and folic acid tablet, information received about birth preparedness, and on key obstetrical danger signs. The content was categorized as “appropriate” if women received the highest quintile of procedures at least once during the last pregnancy that corresponds to eight out of the ten of procedure items received, otherwise “inappropriate”.

### Data collection

The household data were collected via face-to-face interviews. A pre-tested structured questionnaire, developed in the local language (Amharic), was used to collect information about the utilization of the key maternity services (pre-conception and postpartum family planning, antenatal, delivery and postpartum care) and reasons for not seeking maternity care. In addition, health facility assessment was carried out using an observation checklist. The questionnaire and the checklists were developed by reviewing different literature including the WHO guidelines [23, 24], the Ethiopian demographic and health survey [4], and the list of interventions recommended by the Federal Ministry of Health of Ethiopia [25] (**Additional file****1****and Additional file****2**).

For the survey, a total of 20 data collectors and supervisors were deployed after receiving 2 days of intensive training by the principal investigator. The readiness or preparedness of health care facilities to support the provision of maternity (ANC, SBA, PNC) and reproductive health services had been evaluated using the WHO’s criteria [23]. Then the result of each facilities’ readiness score was linked to the individual woman in the corresponding household survey.

### Data analysis and modeling

Data analysis was done using SPSS for windows version 25. We first described the levels of using the key maternal and reproductive services along the continuum. For each of the services, percentages of retention and drop-offs between the successive components along the continuum pathway were computed.

Women living in the same kebele (cluster) may share similar characteristics; hence, the estimates from ordinal logistic regression that assumes all individuals are independent would not be effective. Therefore, by considering the hierarchical structure of the data, where women were nested within the kebeles, a multilevel logistic regression was fitted. This model also enables partitioning of the total variation in the outcome into within-group (in this particular case kebele) and between-group components, which allows in differentiating the relative contributions of level 1 and level 2 variables [26].

For each of the three outcome variables, we fit two multilevel logistic regression models. The first was the empty model that did not contain any explanatory variable. This model was used to determine if our data justified the decision to assess random effects at level 2 (i.e. at kebele level). The second model (the full model) included both level 1 and level 2 variables in addition to cluster (kebele) specific random effects.

During analysis, we used the same groups of predictors for the first and second outcomes. Meanwhile, variables related to services received during antenatal care (the content and timing of first ANC) and modes of delivery were included in the third outcome.

The fixed effects (measures of association) were reported in terms of odds ratios (OR) with their *P*-values and 95% confidence interval (CI) while the results of random effects (measures of variation) were measured using intra-class correlation (ICC) [27]. The ICC was calculated as;

$$ \mathrm{ICC}={\upsigma_1}^2/{\upsigma_1}^2+{\uppi}^2/3. $$

Where: σ_1_^2^ and π2/3 are cluster and individual level variances, respectively. Since there is no separate variance term at Level- l for categorical variables (i.e., the residual variance at level l is fixed to a factor of 1.0), the variance of a logistic distribution is π^2^/3, or approximately 3.29 [26].

## Results

### Socio-demographic characteristics

A total of 1281 (response rate of 95.8%) reproductive-aged women (15–49 years) participated in the household survey. As Table 1 shows, the majority of respondents were married 1208(94.6%), Orthodox Christian followers 1230(96.0%), and Amhara in ethnicity, 1249(97.5%).

**Table 1:** Background characteristics of women who gave births within 12 months preceding the survey date, West Gojjam, Northwest Ethiopia, 2018

Variables	Number of women (***N*** = 1281)	Percentage
Age of the mother (Years)		
15–24	226	17.6
25–34	674	52.6
35–39	291	22.7
> = 40	90	7.0
Residence		
Rural	978	76.3
Urban	303	23.7
Marital Status		
Single	9	0.7
Married	1208	94.3
Divorced	57	4.4
Widowed	7	0.5
Education status		
Cannot read and write	660	51.5
Read and write	164	12.8
Primary education	306	23.9
Secondary Education	101	7.9
Higher Education	50	3.9
Education status of the husband(*n* = 1212)		
Cannot read and write	369	28.8
Read and write	287	22.4
Primary education	379	29.6
Secondary Education	105	8.2
Higher Education	73	5.7
Occupation		
Employed	47	3.7
Merchant	132	10.3
Farmer	886	69.2
Daily worker	88	6.9
Housewife	108	8.4
Others	20	1.6
Occupation of the husband(*n* = 1212)		
Employed	88	6.9
Merchant	161	12.6
Farmer	863	67.4
Daily worker	75	5.9
Others	25	2.0
Religion		
Orthodox	1230	96.0
Catholic	29	2.3
Muslim	19	1.5
Protestant	3	0.2
Ethnicity		
Amhara	1249	97.5
Others	32	2.5
Wealth quintile		
Poorest	60	4.7
Poor	571	44.6
Middle	58	4.5
Rich	336	26.2
Richest	256	20.0
Birth order		
1	288	22.5
2–4	661	51.6
5+	332	25.9
Interval between successive births(*n* = 993)		
< 24 months	46	3.6
24–33 months	245	24.7
34–59 months	630	63.4
> =60 months	72	7.3
Intendedness of the pregnancy		
Intended	1062	82.9
Mistimed	175	13.7
Unwanted	44	3.4
Previous use of family planning		
Yes	942	73.5
No	339	23.5
History of adverse pregnancy outcomes		
Yes	117	9.1
No	1164	90.9
History of Pregnancy related complications		
Yes	135	10.5
No	1146	89.5
Knowledge of at least 2 danger signs		
Yes	388	30.3
No	893	69.5

Over three-quarters, 978(76.3%) of the women were rural residents and almost similar proportion, 993(77.5%) were multi-parous. A high number of grand multiparty, 332(25.9%) was also noted. The age distribution of the participants showed that more than half 674(52.6%) of them were between 25 and 34 years and the mean (+SD) age was 30.3(+ 6.0) years.

Concerning the distribution of the respondents’ educational status, more than half of the women, 660 (51.5%) had never been to school, while only a far smaller proportion, 50 (3.9%) attained tertiary education. Regarding the distribution of the women’s household wealth index almost half, 631(49.3%) of women were belonging to the lowest two wealth quintiles (Table 1).

### Descriptive presentation of key elements of the continuum of maternal care

#### Antenatal care (ANC) follow up

While 898(70.1%) (95% CI: 67.5–72.6%) of the women received antenatal services from skilled health professionals (doctor, health officer, nurse, or midwife in Ethiopian context) at least once, 511(39.9%) (95% CI: 37.2–42.6%) of the women continued for the WHO-recommended four or more ANC visits. Of the mothers who had at least one ANC visit, only134 (14.9%) of them had their first ANC visit during their first trimester, while 615 (68.5%) of them during the second trimester, and the rest 149(16.6%) started during the third trimester.

Furthermore, the findings showed that most of the women who made at least one antenatal care visit did not receive the key ANC service components recommended by the WHO; and only 418 (46.5%) of them received all the items of the antenatal care contents. For instance, of the nine key ANC components considered in this study, a smaller percentage of women had urine 519(57.8%) and blood samples taken 556(61.9%). On the other hand, the coverage of the blood pressure measurement at least once was much better than other antenatal care contents, 857 (95.4%).

#### Use of skilled birth attendant (SBA)

Overall, less than half, 609(47.5%) of the most recent births were assisted by skilled birth attendants either at hospitals or health centers. For those women who gave birth outside the health institutions, sudden onset of labor 395(58.8%) and lack of transportation for getting to health facilities 323(48.1%) were the main barriers mentioned for not seeking care. This is further confirmed by the fact that only 175(28.5%) of the women who delivered in healthcare facilities got an ambulance service to travel to the health facilities when labor started.

The finding further revealed that 178(13.9%) of the women were encountering at least one complication during or immediately after delivery; severe vaginal bleeding 30(16.9%) and prolonged labor 109(61.2%) were the most frequently mentioned problems, among others.

#### Postnatal care use

For the postnatal care, despite 562(43.0%) of the respondents received at least one PNC within 6 weeks after delivery, only a third, 192 (14.9%) of the mothers reported a health check within the first 48 h after birth. Women who reported post-partum care were asked about the contents of care they received; and the most frequently received service was counseling on breastfeeding, reported by 467(83.1%). On the contrary, counseling about follow-ups 154 (27.4%) and postpartum family planning 221(39.3%) were the least frequently received services.

#### Pre-pregnancy and postpartum family planning use

Contraceptive uses, both pre-pregnancy and postpartum, were more commonly reported than any other components of the continuum. Of the respondents, 942(73.5%) reported that they were using modern contraceptives before the index pregnancy. The proportion of women who received modern contraceptives after delivery was 762(59.5%). The contraceptive method mix was dominated by injectable 545 (71.5%) followed by implants, 164(21.5%).

For women who failed to use any modern contraceptive after birth issues related to postpartum amenorrhea 137(26.4%), fear of side effects of hormonal contraceptives 97(18.7%), and infrequent sexual intercourse 119(22.9%) were the topmost reasons cited for non-use of contraception.

### Retention on the continuum of the maternal care pathway

**Fig. 1:**
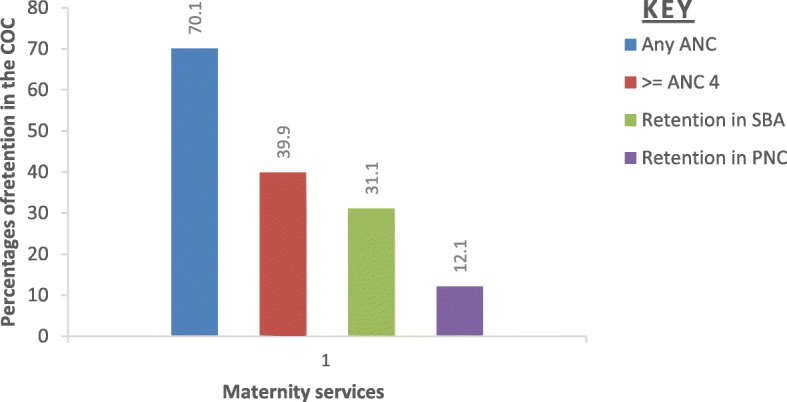
Maternal service utilization along the continuum of maternal care pathway from ANC to SBA and PNC within 48 h after birth in West Gojjam Zone, Northwest Ethiopia, 2018

Figure 1 illustrates the flow of services within the continuum of care to indicate the proportions of women who transit from one maternal service to the next, and the points along the continuum where women drop off from the journey.

As could be seen in the Fig. 1, 70.1%(67.5–72.6%) of the women received ANC services at least once in their pregnancy but a substantial of them(30.2%) did not continue on the pathway to receive 4 or more ANC visits, which was the highest drop-off in the continuum. As a result, only 39.9% (37.2–42.6%) made four or more visits. Across the continuum, the smallest relative drop-off (8.8%) was observed between ANC 4+ and facility delivery; 31.1% (28.5–33.7%) of women who received at least four ANC continued giving birth in health care facilities. Furthermore, after facility delivery about one for every five (19.0%) women did not go on to receive a PNC health check within 48 h of birth (Fig. 1).

Table 2 shows the percentages of women who received the various possible combinations of maternal health services within the continuum of care. These combinations help to point out the closely aligned elements of the continuum of care.

**Table 2:** Percent distribution of reproductive-aged women by different types of maternal health services received for the most recent birth, West Gojjam Zone, Northwest Ethiopia, 2018(*n* = 1281)

S.N	ANC1	ANC 4+	SBA	PNC within48 hrs.	Number (%)
**Not Achieved continuum of maternal care**					
1	No	No	No	No	321(25.1)
	**Partially achieved continuum of maternal care**				
2	Yes	No	No	No	238(18.6)
3	No	No	Yes	No	53(4.1)
4	No	No	No	Yes	–
5	Yes	Yes	No	No	113(8.8)
6	No	No	Yes	Yes	9(0.7)
7	Yes	No	Yes	No	121(9.4)
8	Yes	No	No	Yes	–
9	Yes	Yes	Yes	No	243(19.0)
10	Yes	No	Yes	Yes	28(2.2)
11	Yes	Yes	No	Yes	–
**Fully achieved continuum of maternal care**					
12	Yes	Yes	Yes	Yes	155(12.1)
Total	70.1%	24.5%	47.5%	14.9%	**1281 (100%)**

*Note: Yes = received the service, No = did not receive the service, ANC1 = At least one ANC visit, ANC4 = Four or more ANC visit, SBA = Skilled birth attendant at delivery (or delivery at health centers and hospitals), PNC=Postnatal check-up for the mother within 48 h after birth*

The finding indicated that 321 (25.1%) of women interviewed reported not having any contact at any points of the continuum during their pregnancy, and only 155 (12.1%) accomplished all the stages of the continuum of care (4 + ANC visits, SBA, and PNC visit within 48 h). The result also highlighted that only a few (or none at all) women attended facility delivery or postnatal care within 48 h or both without first having received focused antenatal care, showing positive linkages among different maternal services. Accordingly, facility delivery (SBA) without first having four or more ANC visits was uncommon at less than 5 %. Similarly, PNC follow up was uncommon without having antenatal care visits and delivery assisted by skilled professionals (Table 2).

### Multilevel analysis

As stated in the methods section, three multilevel logit regression models were fitted to identify factors influencing the utilization of maternal health services along the continuum of care.

The first step in the multilevel model analysis was to consider if our data justified the decision to assess random effects at the kebele (cluster) level. In this regard, the result of the empty model revealed that there was a considerable amount of variation for each indicator of maternal health service utilization across the clusters (*P* < 0 .05), which justifies developing a multilevel model.

As shown by the intra-community correlation coefficient (ICC) values, 24.9, 20.1, and 21.2% of the total variances in the use of at least four antenatal cares (ANC4+), facility delivery, and postnatal care within 48 h were attributable to unobserved differences across clusters, respectively (Table 3).

**Table 3:** Parameter coefficients for the multilevel model of retention in the three indicators of maternal healthcare services (ANC, SBA, PNC): empty model, without covariates in West Gojjam Zone, 2018

***Random effect parameter***	ANC4+	***Retention in SBA*** ***(ANC 4+ &SBA)***	***Complete COC*** ***(ANC4+,SBA,& PNC)***
Level-Two variance (95%CI)	1.09*(0.46,2.61)	0.83*(0.34,1.98)	0.89*(0.35,2.18)
**Rho-ICC**	**0.249**	**0.201**	**0.212**
Log-likelihood(−2LL)	4035.857	5515.284	6704.977

Notes: *CI* Confidence interval, *2LL* Log-likelihood, *p < 0.05; ICC (p) = Intra-class correlation Coefficient

When adjusted for all the study covariates, the kebele (Cluster) level variances in the use of ANC 4+, skilled attendance at delivery, and retention in PNC reduced to 22.4, 10.1, and 11.8%, respectively (*p* > 0.05). As shown in Table 4, the full model showed that there are some variations in the predictors of the specific indicators of maternal service use; hence, we present the findings separately for each maternal indicators (Table 4).

### Predictors of ANC 4 + visits

Model-I analyzed the predictors of four or more antenatal care visits by women who took at least one antenatal care service. Results of the adjusted model showed that women’s age, education, and knowledge about pregnancy-related complications were relatively the strongest level-1 predictors. The odds of retention in the antenatal care were higher among mothers aged between 18 and 24 years (AOR = 6.15, 95% CI = 2.41–16.48); women attended at least secondary education (AOR = 3.02, 95% CI = 1.16–7.83); and those who knew at least two pregnancy danger signs of pregnancy (AOR = 3.31; 95%CI; 2.02, 5.41). Moreover, mothers who belonged to the richest wealth tertile (AOR = 2.21, 95% CI = 1.12–4.34) and those who had intended pregnancies (AOR = 2.24, 95% CI = 1.13–4.43) were the other level-1 predictors.

Of level two covariates considered in this study, the highest readiness score of the nearby health care facility (in providing antenatal care) had a positive association with the use of four or more antenatal follow-up (AOR = 2.21, 95%CI; 1.12, 4.34) than women residing near to facilities of low readiness score.

The ICC (rho) value shows a marginal reduction from 24.9% (*p* < 0.05) in the empty model to 22.4% (still appreciably large) in the full model. In other words, the proportion of reduction of variance at the kebele level due to the covariates was only 12.8% ((1.092–0.952)/1.092), indicating continued clustering of antenatal care utilization even after controlling for both level-1 and level-2 covariates (Table 4).

### Retention in skilled birth attendance (ANC4 &SBA)

Model II analyzed the factors associated with the continuation of care from pregnancy to having skilled birth attendance among women who received at least four ANC visits.

In the fully adjusted model, the odds of retention in SBA were higher among ANC clients whose pregnancy was intended (AOR= 6.28, 95% CI 1.56, 25.31); who experienced any kind of pregnancy-related complications (AOR= 2.73, 95% CI; 1.44, 5.19); and those who had better knowledge on key danger signs (AOR = 9.71, 95% CI; 4.56, 20.68) than their counterparts. In addition, women who had attended secondary and above education (AOR = 5.93, 95% CI = 1.92–18.34) and partnered with at least primary education (AOR = 1.63, 95% CI = 1.01–2.64) had a positive association with retention in skilled birth attendance. On the other hand, the age of the women showed an inverse relation with safe delivery; the odds of retention in SBA were seven times higher among ANC clients aged between 15–24 years (AOR = 6.96, 95% CI; 3.33, 14.54) than 35 years and above.

As a level 2 predictor, women who lived in urban areas (AOR = 4.08, 95% CI = 2.63–6.31) were found to be positively associated with receiving skilled attendance at birth. Similarly, women’s odds of giving birth to health facilities was 3.07 (AOR = 3.07, 95% CI; 1.27, 7.41) times higher for women resided near to health facilities that were more ready to deliver antenatal interventions than the women around less ready facilities to antenatal service (Table 4).

### Retention in PNC within 48 h after birth

Model III estimates the effects of predictors on the continuation of care from delivery to early post-delivery period among women who first received at least four antenatal care and skilled birth attendance (i.e. completion of the entire continuum of care).

Retention in the continuum was higher among mothers who initiated ANC within the first 16 weeks (AOR = 7.53, 95% CI; 2.94, 19.29) than those booked lately. In addition, women who received the recommended contents during ANC consultation, which are indications of ANC quality, were virtually three folds [AOR = 3.31, 95% CI; 1.08,10.16) more likely to complete the continuum of maternal care as compared to women received poor quality of antenatal care.

Any history of poor fetal outcomes (e.g. abortion, stillbirth, and low birth weight) and mode of delivery were also important predictors in influencing the utilization of maternal health services. Women who had a history of poor fetal outcome before the last birth were 70 percent (AOR= 0.30, 95%CI; 0.12, 0.79) less likely to use all the maternal services compared to their counterparts. Similarly, the odds of retention in early PNC attendance decreases by 64% for women who delivered spontaneously than assisted deliveries, (AOR=0.36, 95%CI; 0.20, 0.68).

Women married to better-educated husbands were more likely to use facility-based delivery; the odds of retention in the use of postnatal care among women whose husbands were attending secondary or higher education were about three (AOR = 2.48, 95%CI;1.07,5.73) times higher than those married to non-educated husbands. Likewise, housewives were 3.07 times (AOR = 3.07, 95%CI; 0.38, 24.55) more likely to use all maternal services compared to professionally employed women. Women who mentioned at least two pregnancy danger signs were 2.64 (AOR = 2.64; 95%CI; 1.27, 5.49) times more likely to complete the continuum than their counterparts (Table 4).

**Table 4:** Result of multilevel logistic regression for maternal health care service use by women aged 15–49 who had their most recent birth within 12 months preceding the survey, West Gojjam Zone, Ethiopia, 2018

Predictor variables	Adjusted odds ratio (95% CI)					
	ANC 4+		ANC&SBA		ANC,SBA&PNC	
**Fixed Effects**						
**Individual level factors**						
Women’s education						
- Below primary education^a^	**1.00**	**–**	**1.00**	**–**	1.00	… ..
- Primary education	**1.51(1.04,2.22)**	**0.033**^a^	**2.16(1.33,3.49)**	**0.002**^a^	0.92(0.46,1.85)	0.82
- Secondary and above	**3.02(1.16,7.83)**	**0.024**^a^	**5.93(1.92, 18.34)**	**0.002**^a^	1.94(0.33,11.54)	0.46
Husbands education						
- Below primary education	1.00	–	1.00	… ..	1.00	–
- Primary education	1.97(1.34,2.90)	0.001	1.63(1.01,2.64)	0.04^a^	1.01(0.42,2.44)	0.94
- Secondary and above	1.13(0.46,2.74)	^a^0.79	1.58(0.57,4.41)	0.38	**2.48(1.07,5.73)**	**0.005**^a^
Women’s Occupation						
- Employed	1.00	–	1.00	… .	1.00	–
- Farmer	7.35E5(9.49E6,0.001)	0.00	1.03(0.08,14.02)	0.99	0.27(0.10,0.78)	0.015^a^
- Merchant	8.02E5(7.62E-6,0.001)	0.00	2.73(0.18, 42.33)	0.47	3.07(0.38,24.55)	0.29
- House wife	6.30E5(7.57E-6,0.001)	0.00	2.97(0.41, 21.74)	0.28	**3.61(1.08,12.07)**	**0.04**^a^
- Others	0.0001(1.02-E,0.001)	0.00	8.23(0.68, 99.89)	0.10	1.19(0.21,6.69)	0.84
Husband’s Occupation						
- Employed	1.00	–	1.00	…	1.00	–
- Farmer	**0.19(0.01, 2.49)**	**0.20**^a^	0.68(0.21, 2.21)	0.53	5.32(0.95,29.74)	0.06
- Merchant	0.21(0.02,2.72)	0.19	0.60(0.14, 2.56)	0.49	0.92(0.13,6.49)	0.93
- Others	**0.27(0.03,2.70)**	**0.27**	**0.26(0.08, 0.89)**	**0.03**^a^	3.78(0.66,21.62)	0.14
Religion						
- Orthodox Christian	1.00	–	1.00		1.00	–
- Others	0.74(0.33,1.65)	0.48	0.58(0.27,1.27)	0.17	0.42(0.14,1.30)	0.13
Age of the mother						
- 35+ years	1.00.	–	1.00	… … .	1.00	–
- 25–34 years	1.52(0.99, 2.33)	0.052	2.25(1.44, 3.53)	0.001^a^	0.44(0.18,1.06)	0.07
- 15–24 years	**6.15 (2.41, 16.48)**	**0.000**^a^	6.96(3.33,14.54)	0.001^a^	2.33(0.59,9.06)	0.22
Birth interval						
- < 24 months	1.00	–	1.00	–	1.00	–
- 24–33 months	**0.73(0.21, 2.59**)	**0.03**^a^	**0.30(0.12,0.76**)	**0.01**^a^	0.39 (0.08,1.92)	0.25
- 34–59 months	1.25(0.44,3.53)	0.68	0.44(0.18,1.08)	0.07	0.36(0.07,1.91)	0.23
- > = 60 months	2.48(0.77, 8.04)	0.13	1.22(0.45,3.28)	0.69	0.36(0.06,2.35)	0.29
Intendedness of the pregnancy						
- Intended	**2.24(1.13,4.43)**	0.02^a^	**6.28(1.56,25.31)**	0.01^a^	2.09(0.66,6.69)	0.21
- Not Intended	1.00		1.00	… ..	1.00	… …
History of poor fetal outcome						
- Yes	0.99(0.48, 2.06)	0.98	0.87(0.48,1.57)	0.64	**0.30(0.12,0.79)**	0.02^a^
- No	1.00	–	1.00	–	1.00	–
Complications encountered during the last pregnancy						
- Yes	1.23 (0.56,2.70)	0.61	**2.73(1.44, 5.19)**	**0.002**^a^	1.07(0.60,1.89)	0.82
- No	1.00	… …	**1.00**	**–**	1.00	_____
Knowledge on danger signs of pregnancy						
- Knowledgeable	**3.31(2.02, 5.41)**	**0.001**^a^	**9.71(4.56,20.68)**	**0.001**^a^	**2.64(1.27,5.49)**	**0.01**
- Not knowledgeable	**1.00**	**–**	**1.00**	**–**	**1.00**	
Household wealth index						
- Higher	**2.21(1.12,4.34)**	**0.02**^a^	0.98(0.60,1.59)	0.91	0.59(0.29,1.16)	0.13
- Middle	**2.15(1.50,3.09)**	**0.001**	1.27(0.85,1.90)	0.24	0.89()0.52,1.56)	0.70
- Lower	**1.00**	**–**	11.00	–	1.00	–
Timing of first ANC						
- within 16 weeks	–	–	–	–	**7.53(2.94,19.29)**	**0.001**^a^
- After 16 weeks	–	–	–	–	**1.00**	
Content of ANC						
- Appropriate	–	–	–	–	**3.31(1.08,10.16)**	**0.04**^a^
- Inappropriate	–	–	–	–	**1.00**	
Mode of delivery						
- SVD	–	–	–	–	**0.36(0.20,0.68)**	**0.001**
- Assisted/surgery	–	–	–	–	**1.00**	**–**
Community level factors						
Type of residence						
- Urban	1.51(0.83,2.76)	0.18	**4.08(2.63,6.31)**	0.000^a^	1.30(0.53,3.15)	0.57
- Rural	1.00		1.00	–	1.00	… ..
Health facility readiness level						
- High Readiness	**3.53(1.27,9.82**)	0.02^a^	**3.07(1.27,7.41)**	0.021^a^	1.18(0.44,3.16)	0.57
- Medium readiness	2.25(0.68,7.43)	0.18	1.41(0.65,3.06)	0.38	1.49(0.79,2.80)	0.21
- Low readiness	1.00	–	1.00	–	1.00	–
**Random Effects**						
Variance(τ^2^_0)_	0.952(0.56)		0.371(0.26)		0.440(0.39)	
ICC	**0.224**		**0.101**		**0.118**	
-2LL	3259.902		5072.391		1765.477	

. *SVD* Spontaneous vaginal delivery, ^a^ = statistically significant at 0.05′ *ICC* Intra correlation Coefficient;-*2LL* -Log likelihood

## Discussion

The utilization of the three aspects of maternal health care-antenatal, delivery, and postnatal service within 48 h- and the association between individual, household, community, and health facility-related factors have been examined in this study.

The study found that after starting antenatal care visits many women dropped out from the pathway of the continuum and did not have four or more ANC follow-ups, skilled birth attendant or postnatal care within 48 h after birth. Consequently, only 12.1% of the women completed all the three key elements of the continuum of care. This is in line with the findings of similar studies undertaken in some other SSA countries [28, 29]. However, the coverage of CoC was extensively lower than the coverage reported in South Asia [28] and Cambodia [30]. The lower completion rate in the current study compared with other studies might be attributed to the inclusion of only women who received four or more ANC care and PNC within 48 h, whilst others included those who received at least one ANC and PNC within 6 weeks. This large dropout from the continuum of care in our study suggests a higher risk of maternal and neonatal complications, as many women could miss proven interventions at various contact points of the continuum.

As observed in previous studies [31, 32], our finding also re-affirms the noteworthy effect of four or more ANC care for subsequent maternity services as only a few women who did not attend ANC went on to have a facility delivery or PNC check within 48 h. For instance, delivery with an SBA without having received four or more antenatal care was uncommon at less than 1 %. It could be possible to imply that frequent contact in the health system provides a woman the opportunity of getting focused health messages such as birth preparedness and the need to deliver in a health facility. Moreover, based on the effects of using ANC on the subsequent maternal services observed in this study area and elsewhere, it seems right to consider ANC visit as a router that connects the other indicators of maternal health services.

The analytical part of this study identified several factors (operating at various levels.) that have significant effects on the utilization of maternal health care services. The findings have further highlighted that there are commonalities and differences in the factors associated with the use of the three maternal services. Of a set of factors considered in this study, women’s knowledge about the key danger signs of pregnancy remained significant across all three indicators of maternal health services. Women who had a better understanding of the key danger signs of pregnancy were more likely to receive the three maternal services than their counterparts; a finding that reinforces the hypothesis that increased perception of risk encourages the use of care from other studies [33–35]. Health knowledge enables women to be aware of their rights and health status to seek appropriate health services [36].

Mothers’ adherence to the entire continuum of care was higher among those who initiated ANC follow up during the first 4 months of gestation than those booked late. Previous studies from Ethiopia and other SSA countries have also reported the positive correlation between the timing of ANC and subsequent maternity services [10, 11]. Many women, especially in Africa and Asia, hide their pregnancy at the early stage until they had missed several periods before confirming a pregnancy [37], or chose to keep their pregnancy secret until noticed by family members [38]. Further, even if women realized that they are pregnant, the motivation to visit maternity clinics is often superseded by irrational beliefs about pregnancy disclosure [39, 40], especially when women thought that pregnancy has been risk-free. On the other hand, a pregnant woman who did not access antenatal care timely misses opportunities for early detection and prompt treatment of complications, if happen [41, 42].

The health issues of women cannot be addressed without giving due attention to the quality of care for the simple reason that ignoring quality issues affects women’s decisions regarding the time of initiation and continuity of care [43]. Consistent with previous studies [9, 29], our data showed that women who received the key components during ANC consultation, which are indicators for ANC quality, were virtually three times more likely to complete the continuum of care as compared to women received poor quality of antenatal care.

This study has found that the education of a woman and her husband had a significant effect on the utilization of maternal services. These observations are in consonance with previous research finding from Ethiopia [10, 17, 18, 34] and elsewhere [28, 30]. The positive relationship between education and maternal service utilization might be explained as follows. As the level of education increases the social distance between pregnant women and service providers becomes reduced; women become more aware of health-protective information [35], these, in turn, improve women’s ability in accessing the health care services without waiting on the decision made by husbands or other health decisive [44].

The reviewed pieces of literature reported inconsistent findings on the correlation between age and maternal service utilization; some show no effect [45], or older age at motherhood is associated with increased odds of maternity service use [46]. Our finding revealed that younger age at motherhood (between 15 and 24 years) was associated with increased odds of utilizing four or more ANC visits and facility-based delivery than older age women. Our finding is supported by Moyer and colleagues finding that shows an inverse relationship between age and maternity service utilization unless the woman was younger than 18 years [11]. The same study justified that younger mothers may desire to follow modern trends, and be so more likely to use maternal services than older mothers who desire more traditional practices. However, as aged women are more likely to be of high parity and the risk of maternal and fetal complications increase among old and grand multiparous mothers [47], lower levels of retention of these group of women in SBA is particularly problematic.

In this study, unintended pregnancy at the time of conception was associated with less frequent antenatal visits or home deliveries compared to wanted pregnancies. Intentness of pregnancy was also indicated as a factor influencing maternal service utilization in other studies [8, 48, 49]. In this aspect, Yohannes and colleagues from Ethiopian argued that as many women with unintended pregnancy are too young or too old, they may have a negative attitude towards their pregnancies and may go through a period of denial, and hence tend to hide the pregnancy due to fear of stigma [49].

The study also noted that household wealth status was positively and significantly associated with utilization of four or more ANC visits. In effect, the degree of inequalities due to household wealth detected in this study was less than earlier studies in Ethiopia and other developing countries [16, 17, 28]. Unlike other studies, where the effect of household wealth status spans across all maternal service utilization, the effect of household wealth status in this study was limited only for antenatal care use. The lack of variation in delivery and postnatal care services by wealth status in our study might be attributed to the introduction of healthcare financing reforms by the government of Ethiopia, which includes social and community-based health insurance schemes, and charge-free maternity services in public health facilities, among others [50]. Further, for most women labor begins suddenly at night [51], and inaccessibility of transport during this time, accompanied by poor road conditions in remote areas, could have a detrimental effect on SBA than do for antenatal care.

At the community level, urban residence and high level of facilities readiness to antenatal care were found to be associated with higher odds of using facility delivery and antenatal care services, respectively. Consistent with studies in Ethiopia [17, 18, 45] and other countries [20, 28] women who lived in urban areas were more likely to attend an assisted delivery than women residing in rural areas. Lack of infrastructure (e.g. road condition, transport) in rural areas might explain this disparity; in Ethiopia, health facilities are disproportionately distributed in favor of urban residents [52]. In this aspect, Berhan et al. from Ethiopia claimed that several women with obstetric problems paid more than 4000 Ethiopian Birr for less than 100 km travel, which was extremely high by any standard in the country [53]. The urban-rural service gap might also be associated with a low propensity of rural dwellers to seek maternal care during pregnancy; a large proportion of them believe that pregnancy is a natural process requiring no medical intervention [54].

In this study, facility readiness for ANC service was positively associated with the use of frequent ANC visits and facility delivery services. This finding is in agreement with other studies [34, 55]. As mentioned by Barker and his collogues inadequate staffing, lack of drugs, equipment, diagnostics or incompetence of health providers could be barriers to accessing maternal services [56].

Finally, the findings of this study need to be viewed in light of the following limitations. As with other cross-sectional studies, the nature of the data does not allow drawing causal inferences. The data were collected retrospectively and therefore are prone to recall bias. However, to mitigate the effect of recall bias, we focused on the most recent births within 12 months before the survey. Despite the limitations, however, this analysis offers insights into the relationship between services along the continuum of care.

## Conclusions

This study demonstrated that the completion rate of the entire continuum of maternal care was extremely low, indicating that women were not getting the maximum possible health benefit from existing health services. Factors influencing each of the three indicators of maternal care service revealed to operate at various levels - individual, household, community, and health facility, which highlights a need to contextualize efforts. The findings suggest that efforts to promote the use of maternal health services should pay special attention to the needs of old age, uneducated women, rural dwellers, women of unintended pregnancy, and the poor. Further, the positive link between early initiation of first antenatal care with proper content and subsequent maternal care services underscores the need for improving the quality of antenatal care.

## Supplementary information

**Additional file 1.** Questionnaire for household survey

**Additional file 2.** Checklist for health facility survey

## Data Availability

All the data generated or analyzed during this study are included within the manuscript.

## Abbreviations

*ANC*: Antenatal care
*ANC4+*: Antenatal care visits (four or more)
AOR: Adjusted odds ratio
CI: Confidence interval
CoC: Continuum of care
*EDHS*: *Ethiopian* demographic and health surveys
HIV: Human immunodeficiency virus
PNC: Postnatal care
SBA: Skilled birth attendant
SDG: Sustainable development goals
SPSS: Statistical package for social science
SSA: Sub-Saharan Africa
VIF: Variance inflation Factor
WHO: World health organization
