# Supplementary material for: Women’s retention on the continuum of maternal care pathway in west Gojjam zone, Ethiopia: multilevel analysis
Source: BMC Pregnancy Childbirth. 2020 Apr 29;20:258. doi: 10.1186/s12884-020-02953-5 (PMC7191802; doi:10.1186/s12884-020-02953-5)
Supplement: Supplementary file 1 — Additional file 1. Questionnaire for household survey [file 12884_2020_2953_MOESM1_ESM.docx]

### Questionnaire for Kebele Administrators (English Version)

|  | DISTRICT AND COMMUNITY CHARACTERISTICS | | |  | |
| --- | --- | --- | --- | --- | --- |
|  | **Interviewer: This information need to be obtained from kebele administrators or formal delegate. Circle or fill in the blank space for responses according to the nature of the question** | | | | |
| NO | QUESTION AND FILTERS | | RESPONSE and CODE | | SKIP |
| 001 | Questionnaire Id number | | ----------------------- | |  |
| 002 | Name of Woreda (District) | | ----------------------- | |  |
| 003 | Kebele Name | |  |  | |
| 004 | Category of kebele | | 1. Urban 2. Rural |  | |
| 005 | Total number of population in the kebele | | ________________________ |  | |
| 006 | What is the most usual transportation type for the Kebele’s population to reach at the nearest | Hospital | 1. On foot 2. On mules/horseback 3. Vehicle   Others (Specify)………… |  | |
|  | [ONLY ONE OPTION IS POSSIBLE] | Health center | 1. On foot 2. On mules/horseback 3. Vehicle   Others (Specify)………… |  |  |
|  |  | Health post | 1. On foot 2. On mules/horseback 3. Vehicle   Others (Specify)………… |  |  |
| 007 | What do you evaluate the nature of road to health facility? | | 1. Convenient 2. Inconvenient |  | |
| 008 | What is the average distance from center of kebele to main road | | 1. Near 2. Medium 3. Far |  | |

### Women’s Questionnaire (English Version)

***SECTION 1: Household* and Respondent's Background *Characteristics***

| Interviewer : I am going to start by asking you some questions about you and your household  [*Circle the answeramong alternatives OR fill in the blank space]* | | | | | | | |
| --- | --- | --- | --- | --- | --- | --- | --- |
| *Household Characteristics* | | | | | | | |
| S.N | Question | | Response and Code | | | | Skip |
| 101 | How many family members are there in this house hold, including wife and husband? *Include only permanent residents(living greater than 6 months)* | | Family members in number . . . . . . . . … | | | |  |
| 102 | What is the main source of drinking water for members of your household? | | 1. Piped water 2. Dug well 3. Spring 4. River/stream   Others(*Specify*)____________ | | | |  |
| 103 | Where is that water source located? | | 1. In own compound 2. Elsewhere | | | | 105 |
| 104 | How long does it take to go there, get water, and come back? | | Minutes.................................  99. Don’ t know | | | |  |
| 105 | What do you usually do to make the water safer to drink?  RECORD ALL MENTIONED | | 1. Nothing 2. Boiling 3. Add bleach/Chlorine 4. Filter through cloth   Others (Specify)………….. | | | |  |
| 106 | What kind of toilet facility do members of your household usually use? | | 1. No facility but bush/open field 2. Flush toilet 3. pit latrine   Other(specify)_________ | | | |  |
| 107 | What type of fuel does your household mainly use for cooking?  [ MULTIPLE OPTION POSSIBLE] | | 1. Electricity 2. Bio gas 3. Kerosene 4. Charcoal 5. Wood 6. Animal dung   Others (Specify)……………… | | | |  |
| 108 | What type of fuel does your household mainly use for light source?  [ MULTIPLE OPTION POSSIBLE] | | 1. Electricity 2. Bio gas 3. Kerosene lamp 4. Solar light   Others (Specify)……………… | | | |  |
| 109 | Main material of the roof of main house  Record OBSERVATION | | 1. Corrugated iron sheet 2. Thatch/leaf   Other(specify)_______ | | | |  |
| 110 | Main material of floor of main house is made of (observation)  *Write ONLY ONE answer* | | 1. Earth/ mud 2. ceramic tiles 3. cement   other [specify]_________________ | | | |  |
| 111 | Main material of the walls (observation)  *Write ONLY ONE answer* | | 1. wooden and mud 2. stone with lime/cement/ bricks 3. Wood plank   other [specify]________________ | | | |  |
| 112 | What is the primary source of income for this household?  *Circle ONLY ONE answer* | | 1. Farming, including livestock 2. employment/salary 3. petty trading (including sale of fire-wood, charcoal, grass etc) 4. Daily laborer   other [specify]_________________ | | | |  |
| 113 | How many of the following animals does this household own?  IF NONE, RECORD '00'.  [PROBE AND MARK THAT ALL APPLY, MULTIPLE ANSWER IS POSSIBLE] | | Animal type | | | Amount |  |
|  |  |  | Cows/oxen /other cattle | | | _______ |  |
|  |  |  | horses/donkeys/mules | | | ___________ |  |
|  |  |  | Goats/Sheep | | | _________ |  |
|  |  |  | Chickens | | |  |  |
|  |  |  | Bee hives | | |  |  |
| 114 | Does any member of this household own any agricultural land? | | 1. No 2. Yes | | | | 116 |
| 115 | How many hectares/or “Timad” of agricultural land do members of this household own? ***If none, record '00'.*** | | Hectares____________ OR  “Timad”___________ | | | |  |
| 116 | Does any member of this household have an account with a bank/micro finance? | | No (0) | Yes (1) | | |  |
| 117 | Is the house listed as model farmer? | |  | |  | |  |
| 118 | Does your household have the following? | |  | |  | |  |
|  | 119.1 | Radio/ Television |  | |  | |  |
|  | 119.2 | Telephone; landline/ mobile |  | |  | |  |
|  | 119.3 | Bed with cotton/sponges/spring  mattress |  | |  | |  |
|  | 119.4 | An animal-drawn cart/Bicycle/motor  Bike / Bajaj/ car? |  | |  | |  |
|  | 119.5 | Sofa/ chair with Arm or back rest |  | |  | |  |

| Demographic and socio economic characteristics of respondents | | | |
| --- | --- | --- | --- |
| 119 | What is your age?  (WRITE ANSWER IN SPACE) | [________________] years  OR  99. Don’t Know |  |
| 120 | Place of residence | 1. Urban Area 2. Rural Area |  |
| 121 | What is your current marital status? | 1. Single/Never married 2. Married 3. Divorced 4. Widowed | **123** |
| 122 | At what age did you get marry?[INCLUDE ONLY FIRST MARRIAGE] | _______________Years |  |
| 123 | What is your current occupation? | 1. Government Employee 2. Merchant 3. Farmer 4. Daily worker   Others (Specify)_________ |  |
| 124 | What is your partner/husband occupation? | 1. Employee (GO/NGO) 2. Merchant 3. Farmer 4. Daily worker   Others (Specify)_________ |  |
| 125 | What is your religion? | 1. Orthodox 2. Catholic 3. Protestant 4. Muslim   Others (Specify) . . . . . . . . . . . . . |  |
| 126 | What is your ethnicity? | 1. Amhara 2. Agaw 3. Oromo   Others(specify) _________ |  |
| 127 | What is the highest GRADE you completed? | 1. Cannot read and write 2. Read and write 3. Primary education 4. Secondary education 5. Higher education |  |
| 128 | What is the educational level your partner/husband? | 1. Cannot read and write 2. Read and write 3. Primary education 4. Secondary education 5. Higher education |  |

| SECTION TWO: KNOWLEDGE OF WOMEN ABOUT REPRODUCTIVE AND OBSTETRICAL SERVICES | | | | | | | |
| --- | --- | --- | --- | --- | --- | --- | --- |
| No | Question | | | RESPONSE | | | SKIP |
| 201 | Have you heard about optimal birth interval between two consecutive births? | | | 0.No  1.Yes | | | **203** |
| 202 | What is the optimum number of years between two successive births? | | | 1. Below three years  2.Three to five years  3.Above five years  99. I don’t know | | |  |
| 203 | Whom do you think, short birth interval have a health disadvantages, and what? | | | The mother | | Child |  |
|  |  |  |  | 1-None  2-Anemia  3-Bleeding  4-Death  99. Don’t know | | 1-None  2-Low birthweight  3-Preterm baby  4-Death  99. Don’t know |  |
| 204 | After the birth of a child, can a woman become pregnant before her menstrual period has returned? | | | 0.No  1.Yes  99. Don’t know | | |  |
| 205 | Are you aware that exclusive breast feeding in the first 6 months has contraceptive effect? | | | 0.No  1.Yes  99. Don’t know | | |  |
| 206 | Do you know any modern method that women and men can use to delay or avoid pregnancy? | | | 0.No  1.Yes | | |  |
| 207 | When do you think is the appropriate time to begin ANC after amenorrhea? | | | 1.Any Time  2.Within the first 3-4 months  3.Within 5-6 months  4. term of the pregnancy  99.Don’t know  Others (specify)…………… | | |  |
| 208 | How many times [ATLEAST] do you think a women need to go for ANC in a health facility during pregnancy? | | | [ ____________ ] in number | | |  |
| 209 | During her pregnancy period, does a woman need to prepare the following for her delivery? | | | No(0) | Yes(1) | |  |
|  |  | 312.1 | Place of delivery |  |  | |  |
|  |  | 312.2 | Transport |  |  | |  |
|  |  | 312.4 | Money |  |  | |  |
|  |  | 312.4 | Blood donor |  |  | |  |
| 210 | Who is safer assistant for women during delivery? | | | 1.Health professionals (doctor, health officer, nurse or midwife  2.HEW  3.TBA  4.Family/Relative  99. Don’t know  Others (specify)……………… | | |  |
| 211 | Is it culturally acceptable for a woman to leave the house in the first six weeks after she gives birth?  ***Ask, for how many days she is not allowed to leave the household after birth?*** | | | 0.No  1.Yes  99. Don’t know  No of days the mother is expected to stay in home _____ | | |  |
| 212 | Can you tell me what problems that can happen during pregnancy, labor, and after delivery that require immediate attention from a trained health care worker or health facility?    *(MULTIPLE ANSWERS POSSIBLE)* | | | 1. High fever 2. Severe headache /Blurred vision 3. Swelling of hands and face 4. Retained placenta 5. Convulsions/fit/Eclampsia 6. Severe vaginal bleeding 7. Foul smelling discharge 8. Labor >12 hours 9. Mal presentation (if any part of the baby other than the head is seen in the birth passage, like buttocks, hand, foot or cord)   99. Don't know ……………………  Others (Specify:___________________) | | |  |

| SECTION 3 - PRE-PREGNANCY, PRENATAL, INTRAPARTUM, AND POSTNATAL CARE  Interview Read: Now I would like to ask you some questions about your experiences with contraception, pregnancy childbirth and post-delivery care. | | | | | | | | | | | | | | | | | | | | | | | | | | | | | | | | | | | | | | | | | |
| --- | --- | --- | --- | --- | --- | --- | --- | --- | --- | --- | --- | --- | --- | --- | --- | --- | --- | --- | --- | --- | --- | --- | --- | --- | --- | --- | --- | --- | --- | --- | --- | --- | --- | --- | --- | --- | --- | --- | --- | --- | --- |
| No | QUESTION AND FILTERS | | | | | | | | | | | | | | | | | | | | | | | | | | | RESPONSE and CODE | | | | | | | | | | | | |  |
| 301 | How many times have you been pregnant? (Gravida) | | | | | | | | | | | | | | | | | | | | | | | | | | | [_____] times | | | | | | | | | | | | |  |
| 302 | How many live births have you had in your life? (Para) | | | | | | | | | | | | | | | | | | | | | | | | | | | [_________]livebirths: ________Male, ____Female | | | | | | | | | | | | | If she is Gravida I Skip Q#303 |
| 303 | What is the interval between the birth of the last child and the birth of his/her immediate elder child | | | | | | | | | | | | | | | | | | | | | | | | | | | -------- (in months) | | | | | | | | | | | | |  |
| 304 | Did you have history of the following before the current child? ***Remind the mother that this includes not only the history before live baby but also before the current still birth or dead baby after birth, if any*** | | | | | | | | | | | | | | | | | | | | | | | | | | | No(0) | | | | | | | Yes(1) | | | | | |  |
|  |  | | | | | | Previous preterm newborn | | | | | | | | | | | | | | | | | | | | |  | | | | | | |  | | | | | |  |
|  |  |  |  |  |  |  | history of still birth | | | | | | | | | | | | | | | | | | | | |  | | | | | | |  | | | | | |  |
|  |  |  |  |  |  |  | Neonatal death | | | | | | | | | | | | | | | | | | | | |  | | | | | | |  | | | | | |  |
|  |  |  |  |  |  |  | history of abortion | | | | | | | | | | | | | | | | | | | | |  | | | | | | |  | | | | | |  |
|  |  |  |  |  |  |  | Previous newborn low weight | | | | | | | | | | | | | | | | | | | | |  | | | | | | |  | | | | | |  |
|  |  |  |  |  |  |  | Previous cesarean section | | | | | | | | | | | | | | | | | | | | |  | | | | | | |  | | | | | |  |
| 305 | Currently, how many living sons and daughters you have? ***Insert ‘00’ if no child*** | | | | | | | | | | | | | | | | | [_____] sons  AND  [_____] daughters | | | | | | | | | | | | | | | | | | | | | | |  |
| 306 | Number of under five children including the current baby[NAME] | | | | | | | | | | | | | | | | | […………….] | | | | | | | | | | | | | | | | | | | | | | |  |
| 307 | Have you ever used any modern F/P method to delay or avoid getting pregnant of the last baby (NAME)? | | | | | | | | | | | | | | | | | 1. No 2. Yes | | | | | | | | | | | | | | | | | | | | | | |  |
| 308 | At the time you got pregnant with your last baby (NAME), did you want to get pregnant at that time? | | | | | | | | | | | | | | | | | 1. No 2. Yes | | | | | | | | | | | | | | | | | | | | | | |  |
| 309 | If no to Q≠ 308, was your preference to become pregnant then or wait until later? | | | | | | | | | | | | | | | | | 1. I wanted to be pregnant later 2. I didn’t want to be pregnant then or any time in the future | | | | | | | | | | | | | | | | | | | | | | | 312 |
| 310 | If your preference was to wait until later, how long did you prefer to wait? | | | | | | | | | | | | | | | | | 1. Greater or equal to 24 months 2. Less than 24months   99. Don’t Know…………. | | | | | | | | | | | | | | | | | | | | | | |  |
| 311 | What was the reason to become pregnant then while preferring to wait longer than 24 months? | | | | | | | | | | | | | | | | | 1. Lack of awareness 2. Fear of side effect 3. Partner disapproval 4. Because of breast feeding 5. No menstrual resuming   Others(specify)__________ | | | | | | | | | | | | | | | | | | | | | | |  |
|  | | **Antenatal service utilization during the last Pregnancy** | | | | | | | | | | | | | | | | | | | | | | | | | | | | | | | | | | | | | | |  |
| 312 | | Have you ever attended ANC follow up for your current child? [ANC_ANY] | | | | | | | | | | | | | | | | | | | | | | | | 1. No 2. Yes | | | | | | | | | | | | | | | **314** |
| 313 | | If your answer to Q# 312 is no, did the following reasons contribute for none use of your antenatal care? | | | | | | | | | | | | | | | | | | | | | | | | No(0) | | | | | | | Yes(1) | | | | | | | |  |
|  |  | not aware where to go | | | | | | | | | | | | | | | | | | | | | | | |  | | | | | | |  | | | | | | | |  |
|  |  | she thought that it was not necessary | | | | | | | | | | | | | | | | | | | | | | | |  | | | | | | |  | | | | | | | |  |
|  |  | Busy for family care or other domestic work | | | | | | | | | | | | | | | | | | | | | | | |  | | | | | | |  | | | | | | | |  |
|  |  | Husband or other family member were not volunteer | | | | | | | | | | | | | | | | | | | | | | | |  | | | | | | |  | | | | | | | |  |
|  |  | No nearby facility / Facility was too far | | | | | | | | | | | | | | | | | | | | | | | |  | | | | | | |  | | | | | | | |  |
|  |  | Perceived poor quality of maternity Services | | | | | | | | | | | | | | | | | | | | | | | |  | | | | | | |  | | | | | | | |  |
|  |  | concern that a female health worker may not be available | | | | | | | | | | | | | | | | | | | | | | | |  | | | | | | |  | | | | | | | |  |
|  |  | I was healthy | | | | | | | | | | | | | | | | | | | | | | | |  | | | | | | |  | | | | | | | |  |
| 314 | | How many times did you visit the clinic during your pregnancy? | | | | | | | | [ ] times  OR  Don’t remember …………………99 | | | | | | | | | | | | | | | | | | | | | | | | | | | | | | |  |
| 315 | | How many months pregnant were you when you first accessed antenatal care for that pregnancy?  [ ANC_WHEN] | | | | | | | | Months……………………… [___]  Don’t remember …………………99 | | | | | | | | | | | | | | | | | | | | | | | | | | | | | | |  |
| 316 | | Where did you get ANC service?  *[If there is referral linkage please, use the higher health care facility]* | | | | | | | | | | | | | 1. Government Hospital 2. Health center 3. Health post 4. Private clinic 5. Home   Others(Specify)____________________ | | | | | | | | | | | | | | | | | | | | | | | | | |  |
| 317 | | Whom did you see in your last ANC visit? Anyone else?  ***PROBE TO IDENTIFY EACH TYPE OF PERSON AND RECORD THE ONE WHO SUPPORT AT LAST VISIT*** | | | | | | | | | | | | | 1. Doctor 2. Nurse 3. Midwife 4. Health officer 5. Health extension worker   99. I don’t know  Other(Specify)_______________ | | | | | | | | | | | | | | | | | | | | | | | | | |  |
| 318 | | How long (on foot) it takes to reach at that health care facility? **[RECORD THE LAST FACILITY IF VISITED MORE THAN ONE HEALTH INSTITUTE DURING ANC FOLLOW UP]** | | | | | | | | | | | | | _________ Hours  99. I do not know | | | | | | | | | | | | | | | | | | | | | | | | | |  |
| 319 | | What was the average amount of time that you waited to see medical staff when you visited the clinic? | | | | | | | | | | | | | 1. Less than 30 min. 2. 30 min. to 1 hour 3. 1 hour to 1 ½ hours 4. 1 ½ to 2 hours 5. More than 2 hours | | | | | | | | | | | | | | | | | | | | | | | | | |  |
| 320 | | During (any of) your antenatal care visit(s), were you told about the signs of pregnancy complications or danger sign of pregnancy? | | | | | | | | | | | | | 1. No 2. Yes | | | | | | | | | | | | | | | | | | | | | | | | | | 322 |
| 321 | | Which signs of pregnancy complications were you told about?  *PLEASE DO NOT READ LOUD TO RESPONDANTS.*  *PROBE TO TELL YOU MORE*  *MULTIPLE ANSWER IS POSSIBLE* | | | | | | | | | | | | | 1. Vaginal bleeding 2. Vaginal gush of fluid 3. Severe headache 4. Blurred vision 5. Fever 6. Abdominal pain 7. Convulsion   Other (SPECIFY) ……………….... | | | | | | | | | | | | | | | | | | | | | | | | | |  |
| 322 | | Have you encountered ANY of the complications during your previous pregnancy? | | | | | | | | | | | | | | | | | | | | | | No(0) | | | | | | | | | | Yes(1) | | | | | | |  |
|  |  |  | | | | | | | Vaginal bleeding | | | | | | | | | | | | | | |  | | | | | | | | | |  | | | | | | |  |
|  |  |  |  |  |  |  |  |  | Vaginal gush of fluid | | | | | | | | | | | | | | |  | | | | | | | | | |  | | | | | | |  |
|  |  |  |  |  |  |  |  |  | Severe headache | | | | | | | | | | | | | | |  | | | | | | | | | |  | | | | | | |  |
|  |  |  |  |  |  |  |  |  | Blurred vision | | | | | | | | | | | | | | |  | | | | | | | | | |  | | | | | | |  |
|  |  |  |  |  |  |  |  |  | Fever | | | | | | | | | | | | | | |  | | | | | | | | | |  | | | | | | |  |
|  |  |  |  |  |  |  |  |  | Abdominal pain | | | | | | | | | | | | | | |  | | | | | | | | | |  | | | | | | |  |
|  |  |  |  |  |  |  |  |  | Convulsion | | | | | | | | | | | | | | |  | | | | | | | | | |  | | | | | | |  |
|  |  |  |  |  |  |  |  |  | Other(Specify) | | | | | | | | | | | | | | |  | | | | | | | | | |  | | | | | | |  |
| 323 | | During any of antenatal visit were you told about birth preparedness plan?  (Plan for facility delivery, signs and symptoms of labor, items for clean and safe birth such as towel for baby, pajama; discusses about danger signs, transport and fund/money for emergency, identify a potential blood donor and decision-making person and process in case complication occurs at home) | | | | | | | | | | | | 1. No 2. Yes | | | | | | | | | | | | | | | | | | | | | | | | | | | 325 |
| 324 | | Which plans were you told about?  *[DONOT READ LOUD; MULTIPLE RESPONSE IS POSSIBLE]* | | | | | | | | | | | | 1. Plan for facility delivery 2. symptoms of labor 3. discusses about danger signs 4. Emergency transporation 5. Money/emergency fund 6. identify a Potential blood donors   Others(Specify)_____________ | | | | | | | | | | | | | | | | | | | | | | | | | | |  |
| 325 | | At any time before you were pregnant with (BABY'S NAME), how many times did you receive a tetanus injection?  IF NOT TAKEN ANY , RECORD ‘00’  IF 5 OR MORE TIMES, RECORD '5'.  *[Clarify by rephrasing as follows: an injection given to you in the arm or shoulder to prevent the baby from getting tetanus].* | | | | | | | | | | | | | | | | | | | | | | | [____________ ]times  99.Don’t know | | | | | | | | | | | | | | | |  |
| 326 | | When you were pregnant with (BABY'S NAME), how many times did you get a tetanus injection?  IF NOT TAKEN ANY , RECORD ‘00’ | | | | | | | | | | | | | | | | | | | | | | | ____________ Times  99. I don’t know | | | | | | | | | | | | | | | |  |
| 327 | | During the whole pregnancy, for how many days did you take iron/folic acid tablets?  *IF ANSWER IS NOT NUMERIC, PROBE FOR APPROXIMATE NUMBER OF DAYS*  IF NOT TAKEN ANY , RECORD ‘00’ | | | | | | | | | | | | | | | | | | | | | | | [_______________] days  99.Don’t know | | | | | | | | | | | | | | | |  |
| 328 | | During this pregnancy, did you take any drug for intestinal worms? | | | | | | | | | | | | | | | | | | | | | | | 1. No 2. Yes   99.I don’t know | | | | | | | | | | | | | | | |  |
| Now, I would like to ask you about the overall experience of your *ANC* care when you were pregnant with (BABY'S NAME).As part of your antenatal care during this pregnancy, were any of the following done/happened by health providers at least once? (Put“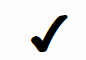”mark) | | | | | | | | | | | | | | | | | | | | | | | | | | | | | | | | | | | | | | | | | |
| 329 | | |  | | | | | | | | | | | | | | | | | No(0) | | | | | | | | | Yes | | | | | | | DK(99) | | | |  | |
|  |  |  | body weight measured | | | | | | | | | | | | | | | | |  | | | | | | | | |  | | | | | | |  | | | |  | |
|  |  |  | Blood pressure measured | | | | | | | | | | | | | | | | |  | | | | | | | | |  | | | | | | |  | | | |  | |
|  |  |  | Urine sample taken | | | | | | | | | | | | | | | | |  | | | | | | | | |  | | | | | | |  | | | |  | |
|  |  |  | Tested for syphilis | | | | | | | | | | | | | | | | |  | | | | | | | | |  | | | | | | |  | | | |  | |
|  |  |  | advised about diet and nutrition during pregnancy | | | | | | | | | | | | | | | | |  | | | | | | | | |  | | | | | | |  | | | |  | |
|  |  |  | Counseling and testing of HIV/AIDS | | | | | | | | | | | | | | | | |  | | | | | | | | |  | | | | | | |  | | | |  | |
|  |  |  | Discussed the importance of F/ planning? | | | | | | | | | | | | | | | | |  | | | | | | | | |  | | | | | | |  | | | |  | |
|  |  |  | Advantages of facility delivery and PNC | | | | | | | | | | | | | | | | |  | | | | | | | | |  | | | | | | |  | | | |  | |
|  |  |  | Discussed on adverse effect of malaria during pregnancy and its prevention(E.g ITN utilization) | | | | | | | | | | | | | | | | |  | | | | | | | | |  | | | | | | |  | | | |  | |
|  | | | **Labor and delivery** | | | | | | | | | | | | | | | | |  | | | | | | | | | | | | | | | | | | | |  | |
| 330 | | | When did you give birth of your last baby | | | | | | | | | | | | | | | | | ________/____/____ | | | | | | | | | | | | | | | | | | | |  | |
| 331 | | | How many months of gestation were for (BABY'S NAME) born? | | | | | | | | | | | | | | | | | ………………….. Months  99. Don’t know | | | | | | | | | | | | | | | | | | | |  | |
| 332 | | | Where did you deliver (name of Baby)?  [PROBE TO IDENTIFY THE TYPE OF INSTITUTION] | | | | | | | | | | | | | | | | | 1. Government Hospital 2. Government Health Center 3. Health Post 4. Private Hospital/Clinic 5. Home   99. Other(Specify:________) | | | | | | | | | | | | | | | | | | | |  | |
| 333 | | | Who decided where you give birth and by whom? | | | | | | | | | | | | | | | | | 1. Myself 2. My husband 3. Both of us 4. Health Development Army 5. My relatives   Other(Specify)___________ | | | | | | | | | | | | | | | | | | | |  | |
|  | | | Ask the following questions, only if a woman has delivered out of health facility | | | | | | | | | | | | | | | | | | | | | | | | | | | | | | | | | | | | |  | |
| 334 | | | Ask : Reasons for not delivering at health care facilities | | | | | | | | | | | | | | | | | | | | | | | | | | | | | | | | | | | | |  | |
|  | | | Do the following attribute for your delivery at home? | | | | | | | | | | | | | | | | | | | | Yes | | | | | | | | | No | | | | | | | |  | |
|  |  |  | Lack of awareness | | | | | | | | | | | | | | | | | | | |  | | | | | | | | |  | | | | | | | |  | |
|  |  |  | Sudden onset of labor | | | | | | | | | | | | | | | | | | | |  | | | | | | | | |  | | | | | | | |  | |
|  |  |  | Husband/family refusal | | | | | | | | | | | | | | | | | | | |  | | | | | | | | |  | | | | | | | |  | |
|  |  |  | Comfortable to give birth in front of TBAs and relatives | | | | | | | | | | | | | | | | | | | |  | | | | | | | | |  | | | | | | | |  | |
|  |  |  | Financial constraints | | | | | | | | | | | | | | | | | | | |  | | | | | | | | |  | | | | | | | |  | |
|  |  |  | Long distance to health Facility | | | | | | | | | | | | | | | | | | | |  | | | | | | | | |  | | | | | | | |  | |
|  |  |  | Inconvenient transport | | | | | | | | | | | | | | | | | | | |  | | | | | | | | |  | | | | | | | |  | |
|  |  |  | Being busy for childcare/Family care | | | | | | | | | | | | | | | | | | | |  | | | | | | | | |  | | | | | | | |  | |
|  |  |  | Poor quality of service | | | | | | | | | | | | | | | | | | | |  | | | | | | | | |  | | | | | | | |  | |
|  | | | Ask the following questions, only if a woman has delivered within a health facility | | | | | | | | | | | | | | | | | | | | | | | | | | | | | | | | | | | | |  | |
| 335 | | | Which mode of transport did you mainly use to reach at the facility?  RECORD ONE RESPONSE ONLY | | | | | | | | 1. Onfoot 2. Ambulance 3. Public transport 4. Cart 5. Traditional stretcher “*kareza”*   Other (specify)……………..…. | | | | | | | | | | | | | | | | | | | | | | | | | | | | |  | |
| 336 | | | Did you go to the facility because there was a problem while you were in labor? or did you plan to deliver your baby in this facility? | | | | | | | | 1. Yes, because there it was planned 2. No, because of problem during labour   99.Don’t know | | | | | | | | | | | | | | | | | | | | | | | | | | | | |  | |
| 337 | | | What was the mode of delivery for your baby? | | | | | | | | 1. Spontaneous vaginal delivery 2. Assisted vaginal delivery 3. C/S | | | | | | | | | | | | | | | | | | | | | | | | | | | | | 342  342 | |
| 338 | | | If delivered through C/S, did you have your cesarean operation plan before you went into labor or decided after your labor had already started? | | | | | | | | 1. Before labor started 2. After labor started   99.Don’t know/can’t remember | | | | | | | | | | | | | | | | | | | | | | | | | | | | |  | |
| 339 | | | Did you or your family pay for services related to delivery? | | | | | | | | Amount | | | | | | | | | | | | | | | DK | | | | | | | | | | | | | |  | |
|  |  |  | Amount of money you or your family pay for services related to delivery?  **If not paid register “00”** | | | | | Transport | | | |  | | | | | | | | | | | | | |  | | | | | | | | | | | | | |  | |
|  |  |  |  |  |  |  |  | For drug and medicine | | | |  | | | | | | | | | | | | | |  | | | | | | | | | | | | | |  | |
|  |  |  |  |  |  |  |  | Accommodation of family | | | |  | | | | | | | | | | | | | |  | | | | | | | | | | | | | |  | |
|  |  |  |  |  |  |  |  | Laboratory | | | |  | | | | | | | | | | | | | |  | | | | | | | | | | | | | |  | |
|  |  |  |  |  |  |  |  | Others (Specify)……… | | | |  | | | | | | | | | | | | | |  | | | | | | | | | | | | | |  | |
| 340 | | | Now I would like to ask you about the overall experience of your delivery care when you were pregnant with (BABY'S NAME). As part of your labor and delivery care, were any of the following done at least once? (Put “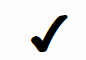” mark) | | | | | | | | | | | | | | | | | | | | | | | | | | | | | | | | | | | | | | |
|  | | |  | | | | | | | | | | | | | | | | | | No(0) | | | | | | | | | | Yes(1) | | | | | | | | DK(99 | | |
|  |  |  | Did providers greet you respectfully | | | | | | | | | | | | | | | | | |  | | | | | | | | | |  | | | | | | | |  | | |
|  |  |  | Did Providers give you information about progress of labor? | | | | | | | | | | | | | | | | | |  | | | | | | | | | |  | | | | | | | |  | | |
|  |  |  | Did adequate privacy given to you during the examination by the health worker | | | | | | | | | | | | | | | | | |  | | | | | | | | | |  | | | | | | | |  | | |
|  |  |  | Were you allowed to have a companion with you during your labor and delivery | | | | | | | | | | | | | | | | | |  | | | | | | | | | |  | | | | | | | |  | | |
|  |  |  | Did the providers encourage to take food or fluid in the process of labor | | | | | | | | | | | | | | | | | |  | | | | | | | | | |  | | | | | | | |  | | |
|  |  |  | Overall, did providers offer compassionate delivery care | | | | | | | | | | | | | | | | | |  | | | | | | | | | |  | | | | | | | |  | | |
| 341 | | | Think about your experience; are you in a position to deliver again in this same facility? | | | | | | | | | | | | | | | | | |  | | | | | | | | | |  | | | | | | | |  | | |
| 342 | | | Did you or your baby experience any complication during or after your delivery**? If yes, write type of problem** | | | | | | | | | | | | | | | | | | | 1. No 2. Yes | | | | | | | | | | | | | | | |  | | | |
| 343 | | | Overall, how do you rate the following types of service in terms of your satisfaction during delivery care at this facility? | | | | | | | | | | | | | | *Satisfied* | | | | | | | | | | *undecided* | | | | | | | | | | | *Dissatisfied* | | | |
|  |  |  | Staff approach | | | | | | | | | | | | | |  | | | | | | | | | |  | | | | | | | | | | |  | | | |
|  |  |  | Waiting time | | | | | | | | | | | | | |  | | | | | | | | | |  | | | | | | | | | | |  | | | |
|  |  |  | Privacy issue | | | | | | | | | | | | | |  | | | | | | | | | |  | | | | | | | | | | |  | | | |
|  |  |  | Charge of services | | | | | | | | | | | | | |  | | | | | | | | | |  | | | | | | | | | | |  | | | |
|  | | | **Postnatal and newborn care** | | | | | | | | | | | | | | | | | | | | | | | | | | | | | | | | | | |  | | | |
| 344 | | | Did you have any postnatal checkup in your last pregnancy? | | | | | | | | | | 1. No 2. Yes | | | | | | | | | | | | | | | | | | | | | | | | | 354 | | | |
| 345 | | | How long after the delivery did the FIRST health check take place? | | | | | | | | | | 1. Less than 24 hours 2. With in 25-48 hours 3. 49-72 hours 4. 73 hours-6 wks 5. > 6 weeks | | | | | | | | | | | | | | | | | | | | | | | | |  | | | |
| 346 | | | How many times you received the service? | | | | | | | | | | ________________Times  99. Donot know | | | | | | | | | | | | | | | | | | | | | | | | |  | | | |
| 347 | | | Where did you get the service | | | | | | | | | | 1. At own home 2. Health post 3. Health center 4. Public hospital 5. Private clinic/hospital   Other(Specify)…………… | | | | | | | | | | | | | | | | | | | | | | | | |  | | | |
| 348 | | | What was your reason for attending health facilities for postnatal care? | | | | | | | | | | 1. I was sick 2. Baby was sick 3. To check my health and the baby’s health 4. during child immunization 5. To get F/ planning   others(specify)_________ | | | | | | | | | | | | | | | | | | | | | | | | |  | | | |
| 349 | | | What was your reason for NOT attending postnatal care? | | | | | | | | | | No(0) | | | | | | | | | | | | | | | | | | | | | | | | | Yes(1) | | | |
|  |  |  | Respondent did not think necessary | | | | | | | | | |  | | | | | | | | | | | | | | | | | | | | | | | | |  | | | |
|  |  |  | The forty days rule, i. e confinement at home | | | | | | | | | |  | | | | | | | | | | | | | | | | | | | | | | | | |  | | | |
|  |  |  | Lack of support person | | | | | | | | | |  | | | | | | | | | | | | | | | | | | | | | | | | |  | | | |
|  |  |  | Husband/family didn’t think necessary | | | | | | | | | |  | | | | | | | | | | | | | | | | | | | | | | | | |  | | | |
|  |  |  | Facility was too far | | | | | | | | | |  | | | | | | | | | | | | | | | | | | | | | | | | |  | | | |
|  |  |  | No transport access | | | | | | | | | |  | | | | | | | | | | | | | | | | | | | | | | | | |  | | | |
|  |  |  | Perceived poor quality | | | | | | | | | |  | | | | | | | | | | | | | | | | | | | | | | | | |  | | | |
|  |  |  | Other(specify | | | | | | | | | |  | | | | | | | | | | | | | | | | | | | | | | | | |  | | | |
|  | | | **For women who delivered at a health facility** | | | | | | | | | | | | | | | | | | | | | | | | | | | | | | | | | | |  | | | |
| 350 | | | How long you waited at the healthcare facility before discharged out? | | | | | | | | | | …………. Hours | | | | | | | | | | | | | | | | | | | | | | | | |  | | | |
| 351 | | | After you gave birth to (NAME OF LAST CHILD), did anyone check on your health while you were still in the facility? | | | | | | | | | | 1. No 2. Yes   99. Do not know/Remember | | | | | | | | | | | | | | | | | | | | | | | | |  | | | |
| 352 | | | Did anyone check on your health after you left the facility? | | | | | | | | | | 1. No 2. Yes   99. Do not know/Remember | | | | | | | | | | | | | | | | | | | | | | | | |  | | | |
| 353 | | | How long after the delivery did the first check take place?***[ Write in hours]*** | | | | | | | | | | ……………. Hours | | | | | | | | | | | | | | | | | | | | | | | | |  | | | |
| 354 | | | Now I would like to ask you about the overall experience of your post-delivery care when you were pregnant with (BABY'S NAME). As part of immediate postnatal care, were any of the following done at least once? (Put “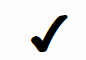” mark) | | | | | | | | | | | | | | | | | | | | | | | | | | | | | | | | | | | | | | |
|  | | | |  | | | | | | | | | | | | No(0) | | | | | | | | | | | | | | Yes(1) | | | | | | | |  | | | |
|  |  |  |  | Asked about the nature of vaginal discharge | | | | | | | | | | | |  | | | | | | | | | | | | | |  | | | | | | | |  | | | |
|  |  |  |  | Advised about breast feeding | | | | | | | | | | | |  | | | | | | | | | | | | | |  | | | | | | | |  |  |  |  |
|  |  |  |  | Advised on diet and nutrition | | | | | | | | | | | |  | | | | | | | | | | | | | |  | | | | | | | |  |  |  |  |
|  |  |  |  | Advised on family planning | | | | | | | | | | | |  | | | | | | | | | | | | | |  | | | | | | | |  |  |  |  |
|  |  |  |  | Informed date of re-check up | | | | | | | | | | | |  | | | | | | | | | | | | | |  | | | | | | | |  |  |  |  |
|  | | | | | **Newborn Care** | | | | | | | | | | | | |  | | | | | | | | | | | | | | | | | | | |  | | | |
| 355 | | | | | How much did your baby (NAME) weigh?  (IF DELIVERED AT HEALTHCARE FACILITIES OR VISITED THE FACILITY WITH IN 7 DAYS AFTER BIRTH ) | | | | | | | | | | | | | _____________K.g  99. Don't know | | | | | | | | | | | | | | | | | | | |  | | | |
| 356 | | | | | What was the gestational age of the child at birth | | | | | | | | | | | | | __________ months  99. Don't know | | | | | | | | | | | | | | | | | | | |  | | | |
| 357 | | | | | What was the condition of the baby at birth? | | | | | | | | | | | | | 1. Apparently normal 2. still birth 3. died after birth 4. Preterm   Others(specify)________ | | | | | | | | | | | | | | | | | | | |  | | | |
| 358 | | | | | If died what was the age before death?  (Record in weeks if less than 1 month) | | | | | | | | | | | | | ________ weeks OR ______months | | | | | | | | | | | | | | | | | | | |  | | | |
| **Post-Partum Family planning** | | | | | | | | | | | | | | | | | | | | | | | | | | | | | | | | | | | | | | | | | |
| 359 | | | | | Since the birth of your child, has your menstruation resumed? | | | | | | | | | | | | | 1. No 2. Yes | | | | | | | | | | | | | | | | | | | | 366 | | | |
| 360 | | | | | When, after birth of the child, does your menses resumed ? | | | | | | | | | | | | | ______________ weeks | | | | | | | | | | | | | | | | | | | |  | | | |
| 361 | | | | | When did you start sexual intercourse after recent birth? | | | | | | | | | | | | | 1. Not started yet 2. Within 7 days 3. 8-14days 4. 15-42 days 5. After 42 days | | | | | | | | | | | | | | | | | | | |  | | | |
| 362 | | | | | Are you currently breasting feeding your child? | | | | | | | | | | | | | 1. No 2. Yes | | | | | | | | | | | | | | | | | | | |  | | | |
| 363 | | | | | Since birth of your last baby, were you visited by a health extension worker HEW) or did any staff member at the health facility counseled you on F/P? | | | | | | | | | | | | | 1. No 2. Yes | | | | | | | | | | | | | | | | | | | |  | | | |
| 364 | | | | | Since birth of your last baby are you or your partner currently using any modern type of family planning? | | | | | | | | | | | | | 1. No 2. Yes | | | | | | | | | | | | | | | | | | | | **367** | | | |
| 365 | | | | | If yes to Q# 369, at what age of the last child did you /your partner start using the family planning method? ***Write in weeks*** | | | | | | | | | | | | | Just at birth  OR  __________________ weeks | | | | | | | | | | | | | | | | | | | |  | | | |
| 366 | | | | | Which method are you/your partner currently using? RECORD ALL MENTIONED | | | | | | | | | | | | | 1. Female sterilization 2. Male sterilization 3. IUCD 4. Injectable 5. Implants 6. Pill   Others (specify)…………… | | | | | | | | | | | | | | | | | | | |  | | | |
| 367 | | | | | If no to Q# 364 What was the reason for not using contraceptive? | | | | | | | | | | | | | 1. Desire to have children 2. Low risk of pregnancy 3. Menstruation is not resumed 4. Husband or partner is not around 5. Opposition from partner   Other___________ | | | | | | | | | | | | | | | | | | | |  | | | |
| 368 | | | | | Does your husband support you in issues related to family planning services? | | | | | | | | | | | | | 1. No 2. Yes | | | | | | | | | | | | | | | | | | | | **370** | | | |
| 369 | | | | | How best does your husband assist you regarding the use of family planning services? | | | | | | | | | | | | | 1. Taking F/P himself 2. Supporting through provision of transport 3. Reminding on dates of appointment 4. Gives items or finances 5. Use of condoms (both male and female condoms)   Any other (specify) … | | | | | | | | | | | | | | | | | | | |  | | | |
| 370 | | | | | What is your Fertility preference? | | | | | | | | | | | | | 1. Want next child Soon 2. Later than 24 months 3. Want no more   99. Unable to decide | | | | | | | | | | | | | | | | | | | |  | | | |
| 371 | | | | | What is your pregnancy status now; pregnant, non- pregnant or do not know? | | | | | | | | | | | | | 1. I am pregnant now 2. Not pregnant   99. Don’t Know………… | | | | | | | | | | | | | | | | | | | |  | | | |
| 372 | | | | | | What is the ideal number of children you want to have[including the existing children] in your life | | | | | | | | | | | | | _____________ | | | | | | | | | | | | | | | | | |  | | | | |
| What are your suggestions for improving maternal health services at government primary health clinics? (Open-ended) ________________________________________________________________________ ________________________________________________________________________ ________________________________________________________________________ ________________________________________________________________________ Checklist for Health Facility Survey | | | | | | | | | | | | | | | | | | | | | | | | | | | | | | | | | | | | | | | | | |
