# Supplementary material for: Women’s retention on the continuum of maternal care pathway in west Gojjam zone, Ethiopia: multilevel analysis
Source: BMC Pregnancy Childbirth. 2020 Apr 29;20:258. doi: 10.1186/s12884-020-02953-5 (PMC7191802; doi:10.1186/s12884-020-02953-5)
Supplement: Supplementary file 2 — Additional file 2. Checklist for health facility survey [file 12884_2020_2953_MOESM2_ESM.docx]

### Checklist for Health Facility Survey

| **Interviewer: Ask the following questions to the person in charge of the facility (manager) or any other concerned body** | | |
| --- | --- | --- |
| INTERVIEWEES BACKGROUND | | |
| 001 | What is your CURRENT position in the health care facility? | …………………………….. |
| 002 | Age | _______________ Years |
| 003 | Sex | 1. Male 2. Female |
| 004 | Qualification | 1. Doctor 2. Health office 3. Nurse 4. Midwife   Others………………... |
| 005 | Total Work experience in years | _______________ Years |

FACILITY IDENTIFICATION

| **SECTION I:** **OVERALL SERVICES IN HEALTH FACILITIES** | | | | | | | | | | | | | | | | | | | | | | |  |
| --- | --- | --- | --- | --- | --- | --- | --- | --- | --- | --- | --- | --- | --- | --- | --- | --- | --- | --- | --- | --- | --- | --- | --- |
| No | QUESTION | | | | | RESPONSE | | | | | | | | | | | | | | | | | Skip |
| 101 | Facility name | | | | | …………………………… | | | | | | | | | | | | | | | | |  |
| 102 | Type of facility | | | | | 1. Hospital 2. Health Center | | | | | | | | | | | | | | | | |  |
| 103 | Location of facility | | | | | 1. Urban 2. Rural | | | | | | | | | | | | | | | | |  |
| 104 | What is the estimated catchment population served by this facility? | | | | | _________ | | | | | | | | | | | | | | | | |  |
| 105 | How many beds are there at this facility (including maternity)? | | | | | _________ | | | | | | | | | | | | | | | | |  |
| 106 | What is the total number of women attending ANC? | | | | | _________ | | | | | | | | | | | | | | | | |  |
| 107 | Number of women delivered at this facility  (in the last 12 months) | | | | | Live birth ___________ Stillbirth___________  Total: _________ | | | | | | | | | | | | | | | | |  |
| 108 | Number of neonatal death within the last 12 months | | | | | _________ | | | | | | | | | | | | | | | | |  |
| 109 | Number of Cesarean sections performed within the last 12 months | | | | | _________ | | | | | | | | | | | | | | | | |  |
| 110 | Number of PNC within six week (42days) for the last 12 months | | | | | _________ | | | | | | | | | | | | | | | | |  |
| 111 | Number of PNC within six week (48 hours) for the last 12 months | | | | | _________ | | | | | | | | | | | | | | | | |  |
| 112 | Number of women who took modern contraceptive in the last 12 months | | | | | _________ | | | | | | | | | | | | | | | | |  |
|  | SECTION II INFRASTRUCTURE | | | | | No (0) | | | | | | | | | | | | | Yes(1) | | | |  |
|  | Basic equipment | | | | |  | | | | | | | | | | | | |  | | | |  |
| 201 | Adult scale | | | | |  | | | | | | | | | | | | |  | | | |  |
| 202 | Child scale | | | | |  | | | | | | | | | | | | |  | | | |  |
| 203 | Thermometer | | | | |  | | | | | | | | | | | | |  | | | |  |
| 204 | Stethoscope | | | | |  | | | | | | | | | | | | |  | | | |  |
| 205 | Blood pressure apparatus (Sphygmomanometer) | | | | |  | | | | | | | | | | | | |  | | | |  |
| 206 | Fetal stethoscope (Fetoscope): | | | | |  | | | | | | | | | | | | |  | | | |  |
| 207 | Gloves( non-sterile) | | | | |  | | | | | | | | | | | | |  | | | |  |
| 208 | Gloves (sterile) | | | | |  | | | | | | | | | | | | |  | | | |  |
| 209 | Vacuum aspiration equipment (MVA) set | | | | |  | | | | | | | | | | | | |  | | | |  |
|  | COMMUNICATION | | | | |  | | | | | | | | | | | | | |  | | |  |
| 210 | Does this facility have a functioning land line telephone  **(CLARIFY THAT IF FACILITY OFFERS 24-HOUR EMERGENCY SERVICES, THEN THIS REFERS TO 24-HOUR AVAILABILITY).** | | | | | No (0) | | | | | | | | | | | | | | Yes(1) | | |  |
| 211 | Does this facility have a functioning cellular telephone or a private cellular phone that is supported by the facility? | | | | |  | | | | | | | | | | | | | |  | | |  |
| 212 | Does this facility have a functioning computer? | | | | |  | | | | | | | | | | | | | |  | | |  |
| 213 | Is there access to internet within the facility today? | | | | |  | | | | | | | | | | | | | |  | | |  |
|  | AMBULANCE/TRANSPORT FOR EMERGENCIES | | | | |  | | | | | | | | | | | | | |  | | |  |
| 214 | Does this facility have a functional ambulance or other vehicle on site for emergency transport for clients that is stationed at this facility or operates from this facility? | | | | | 1. No 2. Yes | | | | | | | | | | | | | | | | | 212 |
| 215 | Does this facility have a functional ambulance or other vehicle for emergency transport for clients that is stationed at another facility or that operates from another facility in near proximity? | | | | | 1. No 2. Yes | | | | | | | | | | | | | | | | | 213  213 |
| 216 | Is fuel for the ambulance or other emergency vehicle available today? | | | | | 1. No 2. Yes | | | | | | | | | | | | | | | | |  |
|  | POWER SUPPLY | | | | |  | | | | | | | | | | | | | |  | | |  |
| 217 | Does your facility have electricity from any source (e.g. electricity grid, generator, solar, or other) including for stand-alone devices (EPI cold chain)? | | | | | 1. No 2. Yes | | | | | | | | | | | | | | | | |  |
| 218 | What is the facility’s main source of electricity? | | | | | 1. Central supply of electricity 2. Generator 3. Solar system   Other (specify) ________ | | | | | | | | | | | | | | | | |  |
| 219 | Other than the main or primary source, does the facility have a secondary or backup source of electricity?  IF YES: What is the secondary source of electricity? | | | | | 1. No secondary source 2. Diesel Generator 3. Solar system   Other (specify) _______ | | | | | | | | | | | | | | | | |  |
|  | BASIC CLIENT AMENITIES | | | | |  | | | | | | | | | | | | | | | | |  |
| 220 | Is water available with in the health facility compound? | | | | | 1. Yes 2. No | | | | | | | | | | | | | | | | |  |
| 221 | What is the most commonly used source of water for the facility at this time? | | | | | 1. Piped into facility 2. Protected dug well 3. Unprotected dug well 4. Protected spring 5. Unprotected spring   Others (specify)………… | | | | | | | | | | | | | | | | |  |
| 222 | Is there a room with auditory and visual privacy available for patient consultations? | | | | | 1. Auditory privacy only 2. Visual privacy only 3. Both auditory and visual 4. Privacy 5. No privacy | | | | | | | | | | | | | | | | |  |
| 223 | Is there a toilet (latrine) in functioning condition that is available for general outpatient client use? | | | | | 1. No 2. Yes | | | | | | | | | | | | | | | | | If No go to 226 |
| 224 | Patient toilet has water for hand washing | | | | | 1. No 2. Yes | | | | | | | | | | | | | | | | |  |
| 225 | Patient toilet has soap for hand washing | | | | | 1. No 2. Yes | | | | | | | | | | | | | | | | |  |
|  | Diagnostic Capacities  Is there any of the following diagnostic facilities in the healthcare facility? | | | | | No(0) | | | | Yes(1) | | | | | | | | | | | | |  |
| 226 | Hemoglobin | | | | |  | | | |  | | | | | | | | | | | | |  |
| 227 | Blood glucose | | | | |  | | | |  | | | | | | | | | | | | |  |
| 228 | Malaria diagnostic capacity | | | | |  | | | |  | | | | | | | | | | | | |  |
| 229 | Urine dipstick-glucose | | | | |  | | | |  | | | | | | | | | | | | |  |
| 230 | HIV diagnostic capacity | | | | |  | | | |  | | | | | | | | | | | | |  |
| 231 | Syphilis RDT | | | | |  | | | |  | | | | | | | | | | | | |  |
| 232 | Urine pregnancy test | | | | |  | | | |  | | | | | | | | | | | | |  |
|  | INFECTION CONTROL: *Observe the HCW providing care for mother in maternity units(ANC, L&D,PNC)* | | | | | Available | | | | Not Available | | | | | | | | | | | | |  |
|  | Please tell me if the following items used for  processing of equipment for reuse are available  and functional in the facility today | | | | |  | | | |  | | | | | | | | | | | | |  |
| 231 | Is there the necessary personal protective barriers for IP practice | | | | |  | | | |  | | | | | | | | | | | | |  |
| 232 | Clean running water (piped, bucket with tap, or pour pitcher) | | | | |  | | | |  | | | | | | | | | | | | |  |
| 233 | Hand-washing soap/liquid soap | | | | |  | | | |  | | | | | | | | | | | | |  |
| 234 | Alcohol based hand rub | | | | |  | | | |  | | | | | | | | | | | | |  |
| 235 | Environmental disinfectant (e.g., chlorine,  alcohol) | | | | |  | | | |  | | | | | | | | | | | | |  |
| 236 | Triple bucket system   - 0.5% Chlorine solution for decontamination - Soapy water for rinsing - Clean water for further rinsing | | | | |  | | | |  | | | | | | | | | | | | |  |
| 237 | Waste receptacle (pedal bin) with lid and plastic  bin liner | | | | |  | | | |  | | | | | | | | | | | | |  |
| 238 | Sharps container ("safety box") | | | | |  | | | |  | | | | | | | | | | | | |  |
| 239 | autoclave | | | | |  | | | |  | | | | | | | | | | | | |  |
| 240 | Electric dry heat sterilize | | | | |  | | | |  | | | | | | | | | | | | |  |
| 241 | Boiler | | | | |  | | | |  | | | | | | | | | | | | |  |
| 242 | Heat source for non-electric equipment | | | | |  | | | |  | | | | | | | | | | | | |  |
| 2243 | How does this facility finally dispose of sharps waste (e.g., filled sharps boxes)? | | | | | 1. Burn incinerator 2. Open burning 3. Dump without burning 4. Remove offsite | | | | | | | | | | | | | | | | |  |
| 2244 | How does this facility finally dispose of medical waste other than sharps boxes? | | | | | 1. Burn incinerator 2. Open burning 3. Dump without burning 4. Remove offsite | | | | | | | | | | | | | | | | |  |
|  | STAFFING | | | | |  | | | | | | | | | | | | | | | | |  |
|  | How many staff with each of the following qualifications are currently assigned to, employed by, or seconded to this facility. For doctors, I would also like to know, of the total number, how many are part-time in this facility | | | | | A) Assigned/ Employed/  Seconded | | | | | | | | | | | | B) Part Time | | | | |  |
| 247 | Generalist (non-specialist) medical doctors | | | | | ______ | | | | | | | | | | | | _______ | | | | |  |
| 248 | Gynecologist and obstetrician | | | | | _________ | | | | | | | | | | | | _________ | | | | |  |
| 249 | Health officer | | | | |  | | | | | | | | | | | |  | | | | |  |
| 250 | Nurses of all categories | | | | |  | | | | | | | | | | | |  | | | | |  |
| 251 | Midwives | | | | |  | | | | | | | | | | | |  | | | | |  |
| 252 | Pharmacist | | | | |  | | | | | | | | | | | |  | | | | |  |
| 253 | Laboratory technicians/technologists | | | | |  | | | | | | | | | | | |  | | | | |  |
| 254 | How many staffs at this facility have received training in reproductive and midwifery skills within the past twelve months?[write “00” if not getting that particular training | | | | | Trained in past 12 months | | | | | | | | | | | | Not trained in the past 12 months | | | | | Remark |
| 254.1 | Emergency obstetric care | | | | |  | | | | | | | | | | | |  | | | | |  |
| 254.2 | Family planning | | | | |  | | | | | | | | | | | |  | | | | |  |
| 254.3 | HIV counseling and testing | | | | |  | | | | | | | | | | | |  | | | | |  |
| 254.4 | Prevention of mother to child transmission of HIV(PMTCT) | | | | |  | | | | | | | | | | | |  | | | | |  |
| 254.5 | Newborn care | | | | |  | | | | | | | | | | | |  | | | | |  |
| 254.6 | Infection prevention | | | | |  | | | | | | | | | | | |  | | | | |  |
|  | INPATIENT AND OBSERVATION BEDS | | | | |  | | | | | | | | | | | | | | | | |  |
| 255 | Of the overnight/inpatient beds in this facility, how many are dedicated maternity beds? THIS DOES NOT INCLUDE DELIVERY BEDS | | | | | # Of dedicated maternity Beds. . . . | | | | | | | | | | | | | | | | |  |
|  | AVAILABLE SERVICES | | | | | No (0) | | Yes (1) | | | | | | | | | | | | | | |  |
| 256 | Does the facility offer the following services every day that facility is open? | | | | |  | |  | | | | | | | | | | | | | | |  |
|  | 256.0 | Antenatal care | | | |  | |  | | | | | | | | | | | | | | |  |
|  | 256.1 | delivery (including normal delivery, basic emergency obstetric care, and/or comprehensive emergency obstetric care) and/or newborn care services | | | |  | |  | | | | | | | | | | | | | | |  |
|  | 256.2 | Postpartum care services (for mother) | | | |  | |  | | | | | | | | | | | | | | |  |
|  | 256.3 | Family planning services | | | |  | |  | | | | | | | | | | | | | | |  |
|  | 256.4 | Post abortion care | | | |  | |  | | | | | | | | | | | | | | |  |
|  | 256.5 | Diagnosis/treatment/counseling for sexually transmitted infections | | | |  | |  | | | | | | | | | | | | | | |  |
|  | 256.6 | Child health services | | | |  | |  | | | | | | | | | | | | | | |  |
|  | 256.7 | VCT on HIV | | | |  | |  | | | | | | | | | | | | | | |  |
|  | 256.8 | PMTCT | | | |  | |  | | | | | | | | | | | | | | |  |
|  | 256.9 | Emergency services and referral | | | |  | |  | | | | | | | | | | | | | | |  |
|  | FAMILY PLANNING SERVICES | | | | |  | |  | | | | | | | | | | | | | | |  |
|  | **Ask the location in the facility where family planning services are provided. Find the person most knowledgeable about family planning services in the facility. Introduce yourself, explain the purpose of the survey and ask the following questions.** | | | | | | | | | | | | | | | | | | | | | | |
| 257 | Are any of the following reproductive health medicines and commodities available in this service site today? ***CHECK TO SEE IF AT LEAST ONE OF EACH MEDICINE/COMMODITY IS VALID (NOT EXPIRED[ENCIRCLE ONE BEST ANSWER]*** | | | Observed  Available | | | | | | | | Not observed | | | | | | | | | | | |
|  |  |  |  |  | | | | | | | |  | | | | | | | | | | | |
| 257.1 | Combined estrogen progesterone oral contraceptive pills | | |  | | | | | | | |  | | | | | | | | | | | |
| 257.2 | Progestin-only contraceptive pills | | |  | | | | | | | |  | | | | | | | | | | | |
| 257.3 | Progestin-only injectable contraceptives | | |  | | | | | | | |  | | | | | | | | | | | |
| 257.4 | Male condoms | | |  | | | | | | | |  | | | | | | | | | | | |
| 257.5 | Female condoms | | |  | | | | | | | |  | | | | | | | | | | | |
| 257.6 | Intrauterine contraceptive device (IUCD) | | |  | | | | | | | |  | | | | | | | | | | | |
| 257.7 | Implants | | |  | | | | | | | |  | | | | | | | | | | | |
| 257.8 | Emergency contraceptive pills | | |  | | | | | | | |  | | | | | | | | | | | |
| 258 | Does this facility provide or prescribe any of the following modern methods of family planning: | | | NO(0) | | | | | | | | YES(1) | | | | | | | | | | |  |
| 258.1 | Male sterilization | | |  | | | | | | | |  | | | | | | | | | | |  |
| 258.2 | Female sterilization | | |  | | | | | | | |  | | | | | | | | | | |  |
| 259 | Please tell me if the following documents are available in the facility today: | | |  | | | | | | | |  | | | | | | | | | | |  |
| 259.1 | National family planning guidelines | | |  | | | | | | | |  | | | | | | | | | | |  |
| 259.2 | Family planning check-lists and/or job-aids | | |  | | | | | | | |  | | | | | | | | | | |  |
|  | PREVENTION OF MOTHER-TO-CHILD TRANSMISSION | | | | | | | | | | | | | | | | | | | | | |  |
|  | FIND THE PERSON MOST KNOWLEDGEABLE ABOUT PMTCT SERVICES IN THE FACILITY. INTRODUCE YOURSELF, EXPLAIN THE PURPOSE OF THE SURVEY AND ASK THE FOLLOWING QUESTIONS. | | | | | | | | | | | | | | | | | | | | | | |
| 260 | As part of PMTCT services, please tell me if this facility provides the following services to clients: | | | | NO(0) | | | | | | YES(1) | | | | | | | | | | | |  |
| 260.1 | Provide HIV counselling and testing services to HIV positive pregnant women for PMTCT | | | |  | | | | | |  | | | | | | | | | | | |  |
| 260.2 | Provide HIV counselling and testing services to infants born to HIV positive pregnant women for PMTCT | | | |  | | | | | |  | | | | | | | | | | | |  |
| 260.3 | Provide ARV prophylaxis to HIV positive pregnant women for PMTCT | | | |  | | | | | |  | | | | | | | | | | | |  |
| 260.4 | Provide ARV prophylaxis to newborns of HIV positive pregnant women for PMTCT | | | |  | | | | | |  | | | | | | | | | | | |  |
| 260.5 | Provide infant and young child feeding counselling for PMTCT | | | |  | | | | | |  | | | | | | | | | | | |  |
| 260.6 | Provide family planning counselling to HIV positive pregnant women for PMTCT | | | |  | | | | | |  | | | | | | | | | | | |  |
| 261 | PMTCT | | | | NO(0) | | | | | | YES(1) | | | | | | | | | | | |  |
| 261.1 | Have you or any provider(s) of PMTCT services: | | | | NO(0) | | | | | | YES(1) | | | | | | | | | | | |  |
| 261.2 | Received any training in PMTCT in the last two years? | | | |  | | | | | |  | | | | | | | | | | | |  |
| 261.3 | Received any training in infant and young child feeding in the last two years? | | | |  | | | | | |  | | | | | | | | | | | |  |
| 261.4 | Is the PMTCT service room or area a private room/area with auditory and visual privacy? | | | | 1. Auditory privacy only 2. Visual privacy only 3. Both auditory and visual privacy 4. No privacy | | | | | | | | | | | | | | | | | |  |
|  | OBSTETRIC AND NEWBORN CARE SERVICES | | | | | | | | | | | | | | | | | | | | | | |
|  | **Find the person most knowledgeable about obstetric and newborn care services in the facility. Introduce yourself, explain the purpose of the survey and ask the following questions** | | | | | | | | | | | | | | | | | | | | | | |
| 262 | Does this facility routinely administer oxytocin injection immediately after birth to all women for the prevention of post-partum hemorrhage? | | | | Yes .……………….. 1  No ………………… 2 | | | | | | | | | | | | | | | | | |  |
| 263 | Please tell me if any of the following interventions for the management of complications during and after pregnancy and childbirth have been carried out in the last 12 months by providers of delivery services as part of their work in this facility | | | | NO(0) | | | | | | | | | | | YES(1) | | | | | | |  |
| 263.1 | Parenteral administration of antibiotics (IV or IM) | | | |  | | | | | | | | | | |  | | | | | | |  |
| 263.2 | Parenteral administration of oxytocic for treatment of post-partum hemorrhage (IV or IM) | | | |  | | | | | | | | | | |  | | | | | | |  |
| 263.3 | Parenteral administration of magnesium sulfate for management of preeclampsia and  eclampsia (IV or IM) | | | |  | | | | | | | | | | |  | | | | | | |  |
| 263.4 | Assisted vaginal delivery | | | |  | | | | | | | | | | |  | | | | | | |  |
| 263.5 | Manual removal of placenta | | | |  | | | | | | | | | | |  | | | | | | |  |
| 263.6 | Removal of retained products of conception | | | |  | | | | | | | | | | |  | | | | | | |  |
| 263.7 | Neonatal resuscitation | | | |  | | | | | | | | | | |  | | | | | | |  |
| 263.8 | Caesarean section | | | |  | | | | | | | | | | |  | | | | | | |  |
| 263.9 | Blood transfusion | | | |  | | | | | | | | | | |  | | | | | | |  |
| 264 | Are the following documents available in the facility today: | | | |  | | | | | | | | | | |  | | | | | | |  |
| 264.1 | National guidelines for Integrated Management of Pregnancy and Childbirth (IMPAC) | | | |  | | | | | | | | | | |  | | | | | | |  |
| 264.2 | Check-lists and/or job-aids for IMPAC | | | |  | | | | | | | | | | |  | | | | | | |  |
| 264.3 | Have you or any provider(s) of delivery services: | | | |  | | | | | | | | | | |  | | | | | | |  |
| 264.4 | Received training in Integrated Management of Pregnancy and Childbirth (IMPAC) in the last two years | | | |  | | | | | | | | | | |  | | | | | | |  |
| 264.5 | Ever received training in newborn resuscitation | | | |  | | | | | | | | | | |  | | | | | | |  |
| 265 | I would like to know if the following basic equipment items are available in this service area today. For each equipment or item, please TRY TO OBSERVE if it is available today and functioning. | | | | A)Available | | | | | | | | | | | B) Functioning | | | | | | |  |
|  |  |  |  |  | Yes | | No | | | | | | | | | Yes | | | | | | No |  |
| 265.1 | Examination light (flashlight ok) | | | |  | |  | | | | | | | | |  | | | | | |  |  |
| 265.2 | Delivery pack | | | |  | |  | | | | | | | | |  | | | | | |  |  |
| 265.3 | Cord clamp | | | |  | |  | | | | | | | | |  | | | | | |  |  |
| 265.4 | Episiotomy scissors | | | |  | |  | | | | | | | | |  | | | | | |  |  |
| 265.5 | Scissors or blade to cut cord | | | |  | |  | | | | | | | | |  | | | | | |  |  |
| 265.6 | Suture material with needle | | | |  | |  | | | | | | | | |  | | | | | |  |  |
| 265.7 | Needle holder | | | |  | |  | | | | | | | | |  | | | | | |  |  |
| 265.8 | Manual vacuum extractor | | | |  | |  | | | | | | | | |  | | | | | |  |  |
| 265.9 | Vacuum aspirator or D&C kit | | | |  | |  | | | | | | | | |  | | | | | |  |  |
| 265.10 | Incubator | | | |  | |  | | | | | | | | |  | | | | | |  |  |
| 265.11 | Disposable latex gloves | | | |  | |  | | | | | | | | |  | | | | | |  |  |
| 265.12 | Blank partograph | | | |  | |  | | | | | | | | |  | | | | | |  |  |
| 265.13 | Blank partograph | | | |  | |  | | | | | | | | |  | | | | | |  |  |
| 265.14 | Delivery bed | | | |  | |  | | | | | | | | |  | | | | | |  |  |
| 265.15 | Resuscitation table (with heat source) (for newborn resuscitation) | | | |  | |  | | | | | | | | |  | | | | | |  |  |
| 265.16 | Newborn bag and mask size 1 for term babies  (for newborn resuscitation) | | | |  | |  | | | | | | | | |  | | | | | |  |  |
| 265.17 | Newborn bag and mask size 0 for pre-term babies (for newborn resuscitation) | | | |  | |  | | | | | | | | |  | | | | | |  |  |
| 265.18 | Electric suction pump (for suction apparatus) | | | |  | |  | | | | | | | | |  | | | | | |  |  |
| 265.19 | Suction catheter (for suction apparatus) | | | |  | |  | | | | | | | | |  | | | | | |  |  |
| 265.20 | Suction bulb, single use (for suction apparatus) | | | |  | |  | | | | | | | | |  | | | | | |  |  |
| 265.21 | Suction bulb, sterilizable multi-use (for suction apparatus) | | | |  | |  | | | | | | | | |  | | | | | |  |  |
| 266 | Are any of the following medicines available in this service site today? **CHECK TO SEE IF AT LEAST ONE OF EACH MEDICINE/COMMODITY IS VALID (NOT EXPIRED)** | | | | Not available | | | | | | | | Available and useable | | | | | | | | | | Available but not useable |
| 266.1 | Antibiotic eye ointment for newborn | | | |  | | | | | | | |  | | | | | | | | | |  |
| 266.2 | Gentamicin injection | | | |  | | | | | | | |  | | | | | | | | | |  |
| 266.3 | Ceftriaxone | | | |  | | | | | | | |  | | | | | | | | | |  |
| 266.4 | Acyclovir | | | |  | | | | | | | |  | | | | | | | | | |  |
| 266.5 | Ampicillin powder for injection | | | |  | | | | | | | |  | | | | | | | | | |  |
| 266.6 | Metronidazole injection | | | |  | | | | | | | |  | | | | | | | | | |  |
| 266.7 | Azithromycin cap/tab or oral liquid | | | |  | | | | | | | |  | | | | | | | | | |  |
| 266.8 | Ciprofloxacin | | | |  | | | | | | | |  | | | | | | | | | |  |
| 266.9 | Benzathine penicillin powder for injection | | | |  | | | | | | | |  | | | | | | | | | |  |
| 266.10 | Benzyl penicilline | | | |  | | | | | | | |  | | | | | | | | | |  |
| 266.11 | Skin disinfectant | | | |  | | | | | | | |  | | | | | | | | | |  |
| 266.12 | Chlorhexidine 4% gel or solution | | | |  | | | | | | | |  | | | | | | | | | |  |
| 266.13 | Betamethasone injection | | | |  | | | | | | | |  | | | | | | | | | |  |
| 266.14 | Dexamethasone injection | | | |  | | | | | | | |  | | | | | | | | | |  |
| 266.15 | Oxytocin injection | | | |  | | | | | | | |  | | | | | | | | | |  |
| 266.16 | Tetanus toxoid | | | |  | | | | | | | |  | | | | | | | | | |  |
| 266.17 | Iron/ Folic acid | | | |  | | | | | | | |  | | | | | | | | | |  |
| 266.18 | Intravenous solution with infusion set  (N/s, RL,DW,DNS) | | | |  | | | | | | | |  | | | | | | | | | |  |
| 266.19 | Oxygen(complete set ) | | | |  | | | | | | | |  | | | | | | | | | |  |
| 266.20 | Calcium gluconate injection | | | |  | | | | | | | |  | | | | | | | | | |  |
| 266.21 | Magnesium sulphate injectable | | | |  | | | | | | | |  | | | | | | | | | |  |
| 266.22 | Antiretroviral drugs(ART) | | | |  | | | | | | | |  | | | | | | | | | |  |
| 266.23 | Insecticide treated bed nets | | | |  | | | | | | | |  | | | | | | | | | |  |
| 266.24 | Anti-malaria drugs (Quinine, chloroquine & other) | | | |  | | | | | | | |  | | | | | | | | | |  |
| 266.25 | Anti-helminthic drugs (mebendazole or others) | | | |  | | | | | | | |  | | | | | | | | | |  |
| 266.26 | Hydralazine injection or Methyldopa or propranolol or any other antihypertensive | | | |  | | | | | | | |  | | | | | | | | | |  |
| 266.27 | Diazepam (injection) | | | |  | | | | | | | |  | | | | | | | | | |  |
| 266.28 | Parenteral oxytocics | | | |  | | | | | | | |  | | | | | | | | | |  |
|  | CESAREAN SECTION(For hospitals only) | | | |  | | | | | | | | | | | | | | | | | |  |
| 267 | CHECK Q# above: | | | | C/S offered | | | | | | | | | | | | | | | | | | C/S not offered |
| 267.1 | Do you have the national guidelines for Comprehensive Emergency Obstetric Care (CEmOC) available in this facility today? | | | | No……………… 0  Yes …………….1 | | | | | | | | | | | | | | | | | |  |
| 267.2 | Have you or any provider(s) of delivery service received any training in Comprehensive Emergency Obstetric Care (CEmOC) in the last two years? | | | | No………………0  Yes ……………1 | | | | | | | | | | | | | | | | | |  |
| 267.3 | Does this facility have a health professional who can perform caesarean section present in the facility or on call 24 hours a day (including weekends and on public holidays)? | | | | No……………… 0  Yes ……………1 | | | | | | | | | | | | | | | | | |  |
| 267.4 | Does this facility have an anaesthetist (or doctor with anaesthetics training) present in the facility or on call 24 hours a day (including weekends and on public holidays)? | | | | No…………… 0  Yes …………1 | | | | | | | | | | | | | | | | | |  |
| LABORATORY SERVICES EQUIPMENT & REAGENTS; Availability of laboratory supplies (5) | | | | | | | | | | | | | | | | | | | | | | | |
| 268 | Which of the following services /or laboratory supplies are availability?  *Request to interview laboratory staff if available.*  *Ask about each service separately* | | Are available at this facility? | | | | | | Provided within the past week? | | | | | | | | | | | | | |  |
|  |  |  | No | | Yes | | | | No | | | | | | | | | | | | Yes | |  |
| 268.1 | Syphilis testing (VDRL/RPR) | |  | |  | | | |  | | | | | | | | | | | |  | |  |
| 268.2 | Blood test—Haemoglobin strip/hematocrit test | |  | |  | | | |  | | | | | | | | | | | |  | |  |
| 268.3 | proteinuria | |  | |  | | | |  | | | | | | | | | | | |  | |  |
| 268.4 | HIV test | |  | |  | | | |  | | | | | | | | | | | |  | |  |
| 268.5 | Pregnancy[HCG strip] test | |  | |  | | | |  | | | | | | | | | | | |  | |  |
| 268.6 | Blood glucose | |  | |  | | | |  | | | | | | | | | | | |  | |  |
| 268.7 | Microscopy or rapid diagnostic test (RDT) for malaria parasites | |  | |  | | | |  | | | | | | | | | | | |  | |  |
| 268.8 | Blood grouping(ABO and Rh) and cross match | |  | |  | | | |  | | | | | | | | | | | |  | |  |
|  | EMERGENCY SERVICES AND REFERRAL | | | |  | | | | | | | | | | | | | | | | | |  |
| 269 | Is a skilled attendant for maternity services available on site, 24 hours/day, 7 days/week? ***[BY SKILLED ATTENDANT INCLUDES A PHYSICIAN, HEALTH*  *OFFICER, MIDWIFE OR NURSE]*** | | | | | | | | | | | | | | | | | | | | | |  |
|  |  | | | | No(0) | | | | | | | | | | | | Yes(1) | | | | | |  |
| 269.1 | Is a skilled attendant for maternity services available [on call or on duty] 24 hours/day, 7 days/week? | | | |  | | | | | | | | | | | |  | | | | | |  |
| 269.2 | Are services for caesarean section available 24 hours/day, 7 days/week? [This is applicable ONLY for hospitals] | | | |  | | | | | | | | | | | |  | | | | | |  |
| 269.3 | Does this facility have a working ambulance or other means of transport in place to refer an obstetric emergency?  [If Yes, specify type of emergency transport system available] | | | |  | | | | | | | | | | | |  | | | | | |  |
| 269.4 | Is the ambulance (or other mode of transport) in working condition at all times? | | | |  | | | | | | | | | | | |  | | | | | |  |
| 269.5 | Is the ambulance (or other mode of transport) in working condition TODAY at this time? | | | |  | | | | | | | | | | | |  | | | | | |  |
| 269.6 | Is the ambulance (or other mode of transport) fueled at all times? | | | |  | | | | | | | | | | | |  | | | | | |  |
| 269.7 | Do facility uses service fees to cover costs of transport? | | | |  | | | | | | | | | | | |  | | | | | |  |
| 269.8 | Do client pay for costs of transport? | | | | 0 No  1 Yes | | | | | | | | | | | | | | | | | |  |
| 270 | To what facility do you USUALLY refer obstetric complications?  How far is that facility, in kilometers? | | | | ______________Health center  OR  ______________ hospital  Don’t know…………  ______________Km | | | | | | | | | | | | | | | | | |  |
| 271 | Does the referral site always have an obstetrician or physician having capacity to do surgery on duty? | | | | 0 No  1 Yes  99 Do not know | | | | | | | | | | | | | | | | | |  |
| 272 | Does the referral site always have a blood bank? | | | | 0 No  1 Yes  99 Do not know | | | | | | | | | | | | | | | | | |  |
| 273 | When you refer a case to another facility how often you get feedback on the outcome of the case? | | | | 0 none  1 Sometimes  2 always | | | | | | | | | | | | | | | | | |  |
|  | Referral registry forms and a referral log sheet are available *(Y/N)* | | | |  | | | | | | | | | | | | | | | | | |  |
|  | GUDELINES | | | |  | | | | | | | | | | | | | | | | | |  |
| 274 | Please tell me if the following guidelines are available in the facility today: | | | | No(0) | | | | | | | | | | Yes(1) | | | | | | | |  |
| 274.1 | Focused ANC checklists and/job aids/ poster | | | |  | | | | | | | | | |  | | | | | | | |  |
| 274.2 | Mg SO4 administration protocol | | | |  | | | | | | | | | |  | | | | | | | |  |
| 274.3 | PMTCT Option B+ desk top reference/pocket guide/job aid, DNA PCR/DBS job aid and HIV  testing algorithm | | | |  | | | | | | | | | |  | | | | | | | |  |
| 274.4 | Infection prevention guideline | | | |  | | | | | | | | |  | | | | | | | | |  |
| 274.5 | Newborn resuscitation flow chart/Helping Babies Breathe Poster | | | |  | | | | | | | | |  | | | | | | | | |  |
| 274.6 | Active management of third stage of labor poster | | | |  | | | | | | | | |  | | | | | | | | |  |
| 274.7 | National newborn case management protocol | | | |  | | | | | | | | |  | | | | | | | | |  |

### 
